# A Hybrid Likelihood Model for Sequence-Based Disease Association Studies

**DOI:** 10.1371/journal.pgen.1003224

**Published:** 2013-01-24

**Authors:** Yun-Ching Chen, Hannah Carter, Jennifer Parla, Melissa Kramer, Fernando S. Goes, Mehdi Pirooznia, Peter P. Zandi, W. Richard McCombie, James B. Potash, Rachel Karchin

**Affiliations:** 1 Department of Biomedical Engineering and Institute for Computational Medicine, Johns Hopkins University, Baltimore, Maryland, United States of America; 2 Stanley Institute for Cognitive Genomics, Cold Spring Harbor Laboratory, Cold Spring Harbor, New York, United States of America; 3 Department of Psychiatry and Behavioral Sciences, Johns Hopkins School of Medicine, Baltimore, Maryland, United States of America; 4 Department of Psychiatry, University of Iowa, Iowa City, Iowa, United States of America; Wellcome Trust Sanger Institute, United Kingdom

## Abstract

In the past few years, case-control studies of common diseases have shifted their focus from single genes to whole exomes. New sequencing technologies now routinely detect hundreds of thousands of sequence variants in a single study, many of which are rare or even novel. The limitation of classical single-marker association analysis for rare variants has been a challenge in such studies. A new generation of statistical methods for case-control association studies has been developed to meet this challenge. A common approach to association analysis of rare variants is the burden-style collapsing methods to combine rare variant data within individuals across or within genes. Here, we propose a new hybrid likelihood model that combines a burden test with a test of the position distribution of variants. In extensive simulations and on empirical data from the Dallas Heart Study, the new model demonstrates consistently good power, in particular when applied to a gene set (*e.g.*, multiple candidate genes with shared biological function or pathway), when rare variants cluster in key functional regions of a gene, and when protective variants are present. When applied to data from an ongoing sequencing study of bipolar disorder (191 cases, 107 controls), the model identifies seven gene sets with nominal p-values
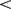
0.05, of which one MAPK signaling pathway (KEGG) reaches trend-level significance after correcting for multiple testing.

## Author Summary

Inexpensive, high-throughput sequencing has transformed the field of case-control association studies. For the first time, it may be possible to identify the genetic underpinnings of complex diseases, by sequencing the DNA of hundreds (even thousands) of cases and controls and comparing patterns of DNA sequence variation. However, complex diseases are likely to be caused by many variants, some of which are very rare. Taken one at a time, the association between variant and disease phenotype may not be detectable by current statistical methods. One strategy is to identify regions where important variants occur by “collapsing” variants into groups. Here, we present a new collapsing approach, capable of detecting subtle genetic differences between cases and controls. We show, in extensive simulations and using a benchmark set of genes involved in human triglyceride levels, that the approach is potentially more powerful than existing methods. We apply the new method to an ongoing sequencing study of bipolar cases and controls and identify a set of genes found in neuronal synapses, which may be implicated in bipolar disorder.

## Introduction

Research efforts over the past few years have yielded an explosion of exome sequencing studies and exomic variation data (reviewed in [1], [2]). One surprising result has been the discovery of hundreds of thousands of novel and rare nonsilent variants in protein coding genes, some of which may have functional consequences related to human health. Common diseases, once hypothesized to be primarily due to common variants [3], are now believed to have heterogeneous genetic causes, due to both common and rare variants [4]–[6].

These developments have created demand for a new generation of statistical and informatics approaches. Increasingly powerful analysis methods have been developed to enable detection of association between phenotype and variants with small to moderate effect sizes (reviewed in [6]). Rather than testing each variant individually, variants can be collapsed or summed with a “burden” approach, in which the strength of phenotypic association is considered with respect to a group of variants occurring at a common region or allelic frequency threshold [7]–[10]. The contribution of each variant to the association may be weighted by frequency or bioinformatically predicted impact [10]. Burden strategies yield a power gain, compared to independent tests of single variants, but they lose power when variants with a neutral or protective effect are included. Regression models [11] and overdispersion tests [12] have been designed to detect variants that affect phenotype, regardless of the direction of the effect (deleterious or protective). New approaches continue to be introduced, such as a mixture model that incorporates gene-gene interactions and an adaptive weighting procedure [13]. A recent study has even suggested that single-variant test statistics may be more powerful than collapsing strategies on real data [14]. Importantly, no single method appears to be superior for all phenotypes, genomic regions, disease models, and populations [6], [15], [16].

Here we describe a new hybrid likelihood test BOMP (Burden Or Mutation Position test), designed for case-control exome sequencing studies, to detect the presence of causal variants in a functional group. The functional group can be defined as a gene, genomic region, or gene set (multiple genes involved in a pathway or biological process). The test can incorporate variant weighting by bioinformatically-predicted functional impact. We combine, into a single statistic, a directional burden test in which low frequency variants have increased weight and a non-directional position distribution test that does not consider allele frequency. Our burden test uses a collapsing strategy and metrics of variant functional importance, which are similar to previously published burden tests (Table S1). An advantage of our test is that its formulation into a likelihood ratio uniquely allows us to combine it with the position distribution test. The two tests complement each other and together yield increased power to detect biologically important variants, particularly when applied to a gene set containing genes with different kinds of variants (*e.g.*, rare, low frequency, common, protective).

To assess the utility of BOMP, we compare its power to three leading methods for variant case-control association testing: VT [10] a mutation burden statistic, SKAT [12] a regression model and overdispersion test, and KBAC [13], which uses mixture modeling and kernel density estimation. We generate simulated case-control studies ranging from 200 to 5,000 individuals, using two demographic growth models, and eight *disease etiologies* (models of disease causation). We also apply BOMP to dichotomized empirical data from a study of quantitative traits, the Dallas Heart Study, which investigated the association between variants in angiopoietin-like (ANGPTL) proteins and triglyceride metabolism [17].

In these experiments, BOMP is consistently powerful across a spectrum of disease causality models, in simulations of case-control studies drawn from populations of African-American and European-American individuals, and for the ANGPTL variants from the Dallas Heart Study. It appears to be particularly useful for detecting genes containing causal variants when protective variants are present, when a disease phenotype is associated with variants that cluster in key regions on a gene, when a causal variant is common, or when applied to a candidate gene set, rather than a single candidate gene.

Finally, we apply BOMP to identify causal gene sets in an an ongoing, whole-exome case-control sequencing study of bipolar disorder. We find that seven gene sets are nominally associated with bipolar disorder and that one “MAPK signaling pathway” (KEGG) trends towards significance after correcting for multiple gene sets tested. Notably, this pathway has been implicated previously in bipolar disorder [18].

## Results

We evaluated the power of the BOMP hybrid likelihood model with both simulations and empirical data from the Dallas Heart Study [17]. All results were compared to several leading statistical methods to detect causal variation in case-control association studies. We attempted to select representative methods for burden, regression, and mixture modeling approaches.

First, we assessed the power of BOMP to detect genes with causal variants in an extreme phenotype case-control study, for a disease with 1% population prevalence, and significance level 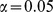. We considered that deleterious causal variants might either be rare, low frequency or common and that modifying protective variants might be present. Power to detect causal variants was assessed initially with respect to a single candidate gene and then for candidate gene sets, ranging in size from 2 to 24 genes. We studied gene sets in which all genes contained causal variants and those in which only a fraction of genes contained causal variants. Both African-American and European-American demographic models were considered. For each combination of attributes (disease etiology, population demographic, case-control study size), 250 case-control studies were simulated to assess power.

### Power analysis of simulated case-control studies

**Figure 1:**
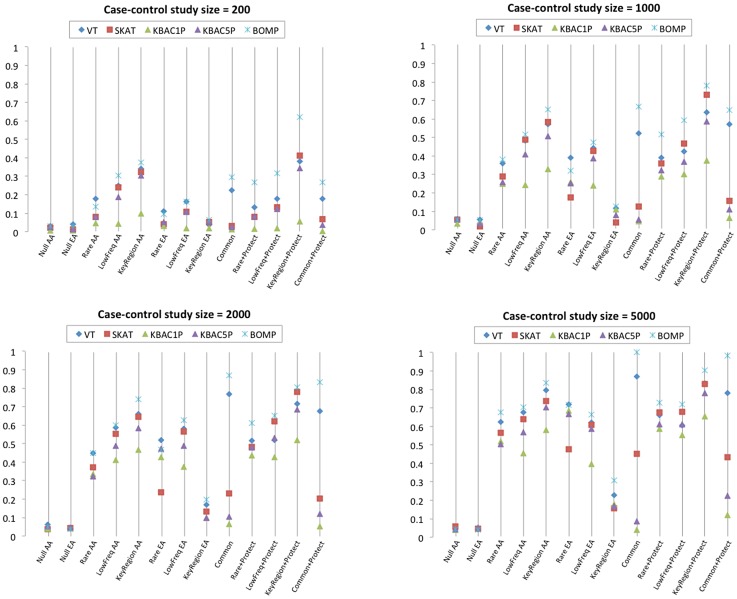
Single gene methods power comparison. Power estimates for BOMP, VT, SKAT, KBAC (KBAC1P = minor allele frequency defined as 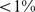, KBAC5P = minor allele frequency defined as 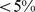). Each vertical line represents power estimates for each method, based on 250 simulated case-control studies. AA = the case-control studies were drawn from gene populations generated with an African-American simple bottleneck demographic model. EA = the case-control studies were drawn from gene populations generated with a European-American exponential growth demographic model. The eight variant causality (disease etiology) models are defined in Table 1. Since the European-American demographic model does not account for common or protective variants, etiologies involving common or protective variants were only considered for the African-American demographic model.

In single-gene case-control study simulations, a study size of 2000 (1000 cases, 1000 controls) was required for any of the methods to achieve at least 80% power to detect causal variants. BOMP had 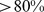 power for three of the tested disease etiologies (Common variant, KeyRegion+Protect, and Common+Protect). When the study size was increased to 5000 (or 10000 (Figure S1)), several of the methods (BOMP, SKAT, VT, and KBAC5P (MAF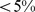)) had 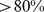 power for selected etiologies (Figure 1). BOMP was consistently more powerful than other methods and appeared to be particularly useful for certain disease etiologies (Key region variant, Common variant, and all etiologies involving protective variants (Table 1)). All methods were less powerful when applied to case-control studies using the European-American demographic model (in which variants are either rare or singletons) (Figure S2).

**Table 1:** Eight disease etiologies used in simulation experiments.

Disease Etiology Name	MAF Deleterious^1^	Selection Coeff Deleterious^2^	Effect size Deleterious^3^	Selection Coeff Protective^4^	Effect size Protective^5^	Variant Functional Role^6^	Demographic model(s)
Rare variant	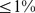	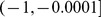	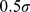	NA	NA	NS	AA,EA
Low frequency variant	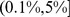	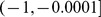	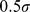	NA	NA	NS	AA,EA
Key region variant	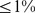	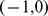	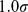	NA	NA	NS	AA,EA
Common variant	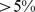	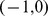	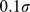	NA	NA	NS	AA
Rare+Protect	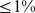	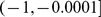	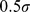	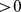	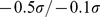 *	NS	AA
LowFreq+Protect	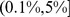	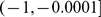	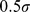	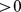	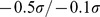	NS	AA
KeyRegion+Protect	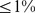	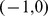	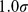	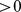	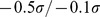	NS	AA
Common+Protect	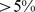	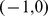	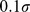	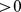	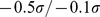	NS	AA

*Rare variant* = disease caused by multiple rare deleterious variants. *Low frequency variant* = disease caused by multiple low frequency deleterious variants. *Key Region variant* = rare deleterious variants are localized to key regions. *Common variant* = disease caused by a single deleterious common variant. The etiologies *Rare+Protect*, *LowFreq+Protect*, *KeyRegion+Protect* and *Common+Protect* were identical to the first four except that they include protective variants.

1 Minor allele frequency of deleterious causal variants,

2 Selection coefficients of deleterious causal variants,

3 Effect size of deleterious causal variants,

4 Selection coefficient of protective causal variants,

5 Effect size of protective modifier variants,

6 Required functional role of causal and protective variants, NS = coding non-synonymous, AA = African-American simple bottleneck demographic model [44], EA = European-American exponential growth demographic model [19]).

*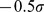 for protective modifier variants with AF
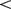
5%, 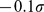 for protective modifier variants with AF
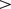
5%.

Next, we explored how the power of the tested methods could be improved by application to a candidate gene set rather than a single candidate gene. We simulated case-control studies, in which each genomic individual had multiple genes, all or some of which contained causal variants. The gene sets in which all genes contained causal variants ranged from 2 to 5 genes. Gene sets with mixtures of casual and non-causal genes ranged from 4 to 15 genes (ratios of causal to non-causal 3∶1, 3∶3, 3∶6, 3∶9, and 3∶12). Causal variants were equally likely to be from any of the disease etiologies dominated by rare variants. The assumption that even 25% of genes in a set contain causal variants is certainly optimistic, but this experiment allowed us to compare the extent to which each method was affected by the fraction of causal genes in a set.

**Figure 2:**
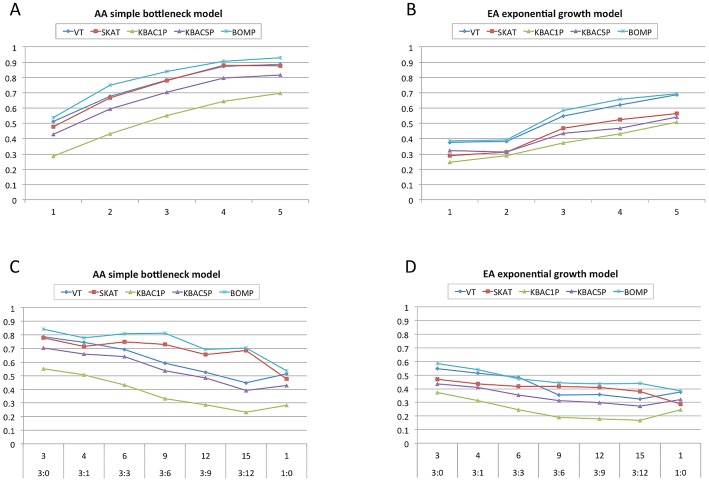
Power estimates for multiple gene case-control studies with causal variants equally likely to be from any disease etiology dominated by rare variants. A,B. X-axis shows number of candidate genes in 250 simulated case-control studies (approximately one-third each from disease etiologies Rare, LowFreq and KeyRegion). All genes contain causal variants. For each method, average power is shown. Power increases for all methods as the number of candidate genes with causal variants increases. C,D. X-axis shows the number of candidate genes and the ratio of genes containing causal variants to those that do not contain causal variants. As the ratio decreases, the power of the tested methods also decreases. (Tested methods are BOMP, VT, SKAT and KBAC1P = minor allele frequency defined as 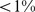, KBAC5P = minor allele frequency defined as 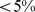). AA = the case-control studies were drawn from gene populations generated with an African-American simple bottleneck demographic model. EA = the case-control studies were drawn from gene populations generated with a European-American exponential growth demographic model.)

When all genes in a gene set contained causal variants, power increased for all methods as gene set size increased. When the gene sets contained a mixture of genes, both with and without causal variants, the power decreased with the causal to non-causal ratio. For the African-American demographic model, BOMP and SKAT were the most robust to gene sets with low causal to non-causal ratio. As in the single gene experiment, all methods had less power in the European-American demographic than in the African-American. For the European-American, none of the methods had power 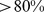 for any of the gene sets. For the African-American demographic, BOMP, SKAT, and VT had power 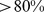 when gene sets of sizes 4 and 5 contained all causal variants. BOMP was the only method with power 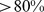 for any of the mixed gene sets tested (gene set sizes 3,6, and 9, with ratio of causal to non-causal 3∶0, 3∶3 and 3∶6) (Figure 2).

**Figure 3:**
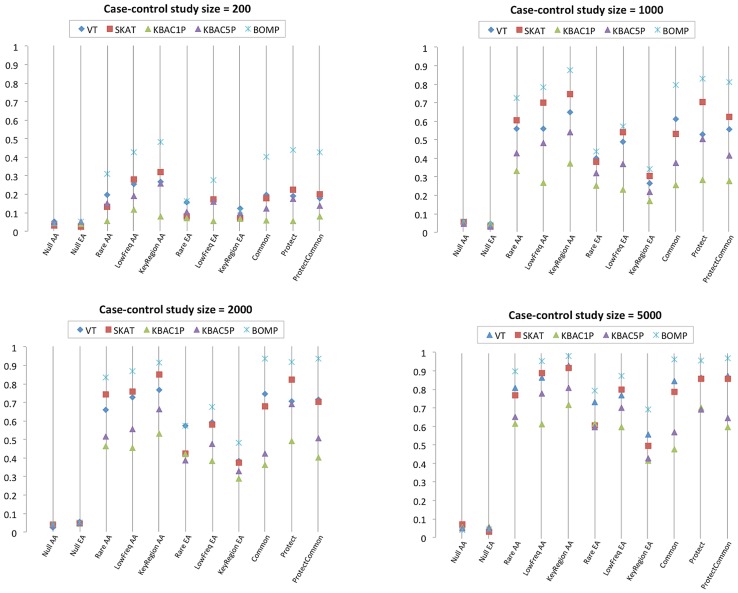
Power estimates for multiple genes case-control studies with causal variants from disease etiologies randomly sampled from nine multinomial distributions (Figure S3). Power estimates for BOMP, VT, SKAT, KBAC (KBAC1P = minor allele frequency defined as 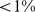, KBAC5P = minor allele frequency defined as 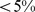). Each vertical line represents power estimates for each method, based on 250 simulated case-control studies. The genomic individuals each had nine genes, of which three contained causal variants and six did not. The disease etiologies for the three genes with causal variants were randomly sampled from nine multinomial distributions (Figure S3). AA = African-American simple bottleneck demographic model. EA = European-American exponential growth demographic model.

Next, we reconsidered the assumption that casual variants in a gene set were equally likely to come from a few disease etiologies. Instead, we sampled disease etiologies from nine multinomial distributions (Figure S3). For these experiments, the number of candidate genes was fixed at nine and the ratio of causal to non-causal genes was 3∶6. BOMP's power advantage over the other tested methods was larger in this experiment than in the single candidate gene experiment. For case-control study size of 1000, BOMP power was 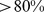 for the multinomial distributions dominated by the key region variant etiology (African-American) and etiologies involving protective variants. For case-control study size of 2000, BOMP power was 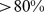 for all six multinomial distributions possible for the African-American model (Figure 3), and SKAT power was 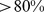 for the multinomial distributions dominated by the key region variant etiology (African-American) and etiologies involving protective variants.

**Figure 4:**
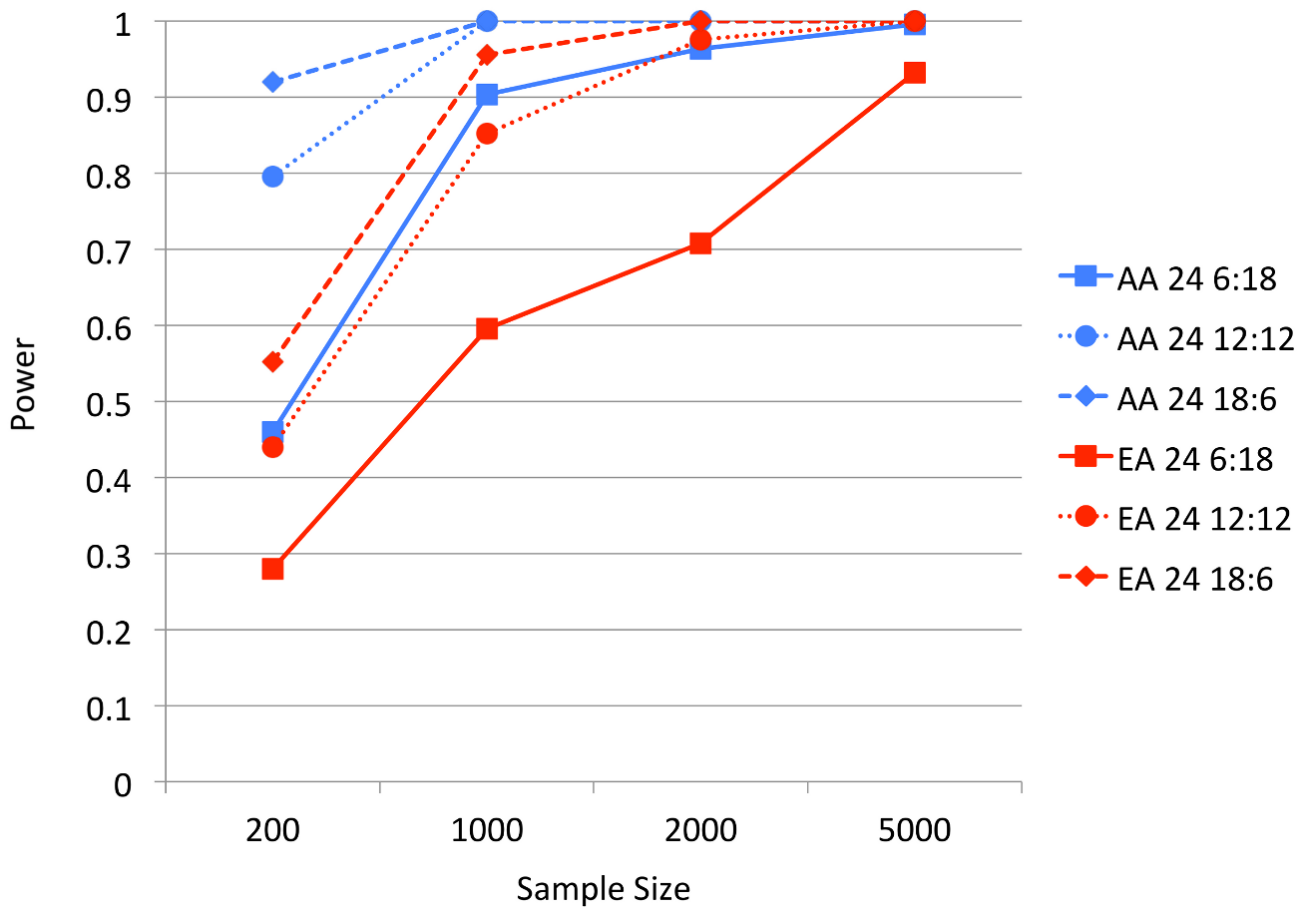
BOMP Power estimates for multiple genes (24) case-control studies. Power estimates for BOMP; each estimate is based on 250 simulated case-control studies ((approximately one-third each from disease etiologies Rare, LowFreq and KeyRegion). The genomic individuals each had 24 genes, the ratio of genes with causal variants to those without causal variants was either 1∶3 (6 causal, 18 non-causal), 1∶1 (12 causal, 12 non-causal), or 3∶1 (18 causal, 6 non-causal). AA = African-American simple bottleneck demographic model. EA = European-American exponential growth demographic model.

We explored the power of BOMP with respect to case-control study size, using a set of 24 candidate genes as the functional group. We varied the ratio of casual to non-causal genes from 1∶3, 1∶1, and 3∶1. Here, causal variants were again equally likely to be from any of the disease etiologies dominated by rare variants. For a case-control study size of 1000, BOMP's power exceeded 0.8, regardless of the causal-to-non-causal gene ratio (African-American only), and for the 1∶1 and 3∶1 causal-to-non-causal gene ratios for European-American. A study size of 200 was sufficient for power 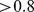 for 1∶1 and 3∶1 ratios (African-American only) (Figure 4).

We reasoned from these results that, for a population whose allele frequency spectrum is similar to our European-American demographic model simulations, current whole-exome case-control studies are not sufficiently powered. These studies lack power to find causal variants both at the single gene level (as proposed by [19]) and for modestly-sized gene sets. However, if the allele frequency spectrum is more similar to the African-American demographic model, BOMP may be able to detect causal variation in larger gene sets, given the size of current whole-exome studies.

To test this hypothesis, we considered a case-control study of 200 individuals, using a set of 100 candidate genes as the functional group. The disease etiologies of causal genes were sampled from three categories with the ratio of 10 (Rare variant or Low frequency variant or Key region variant) :1 (Common variant) : 1 (any etiology involving protective variants). Etiologies were sampled with equal probability within each category. As before, we varied the ratio of causal to non-causal genes in the set from 1∶3, 1∶1, 3∶1. BOMP power was 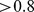 for all scenarios (0.852 for 1∶3, 0.956 for 1∶1 and 0.956 for 3∶1).

### Relative contributions of mutation burden and mutation position distribution in simulated case-control studies

**Figure 5:**
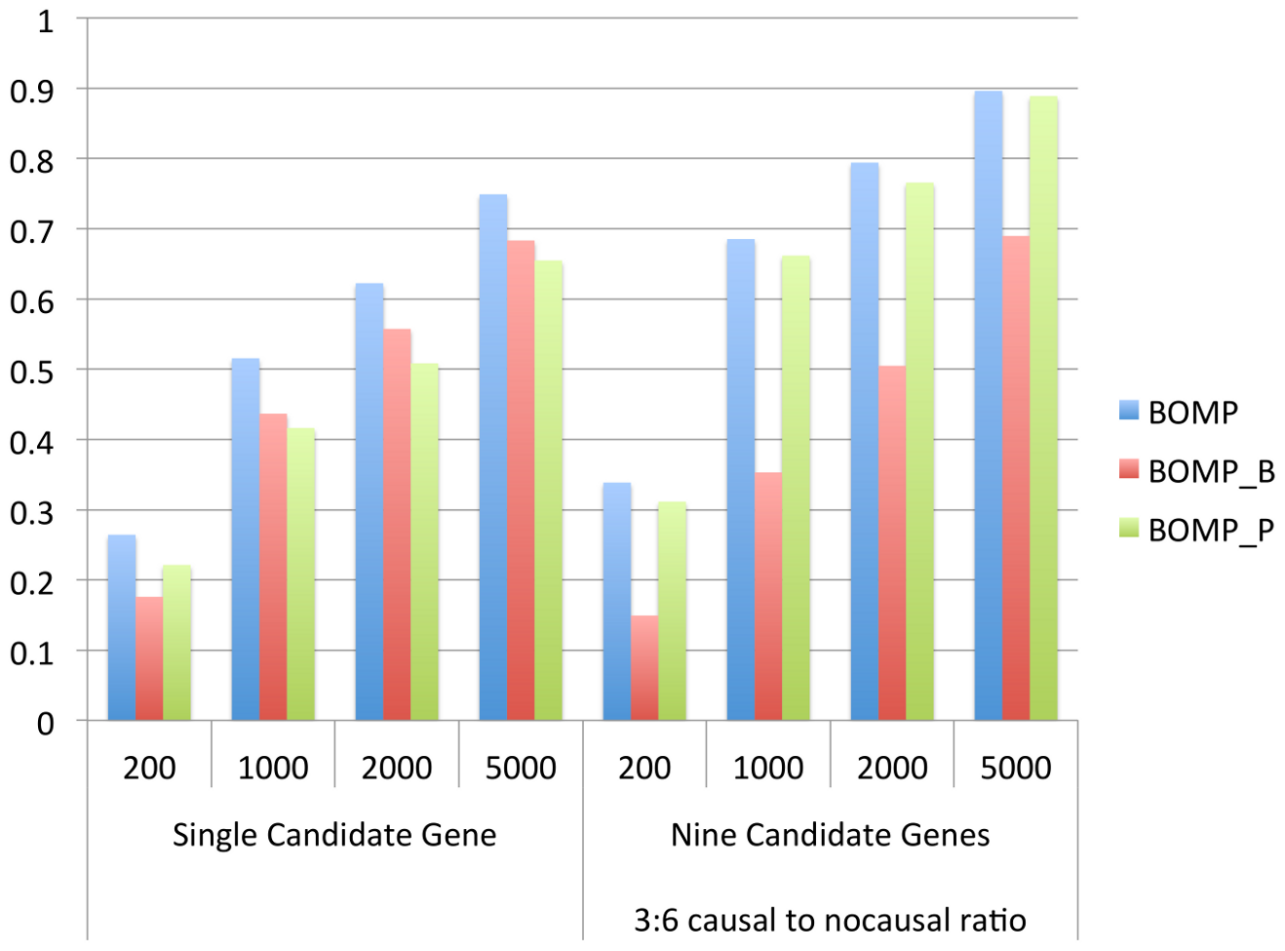
BOMP burden and position statistics complement each other. Breakdown of contribution of BOMP mutation burden (BOMP_B) and BOMP position distribution (BOMP_P) statistics averaged over single candidate gene power estimates (Figure 1) and multiple candidate gene power estimates (nine genes, 3 with causal variants and 6 with no causal variants) (Figure 3) for case-control study sizes of 200, 1000, 2000, and 5000. Combining the two statistics consistently yielded improved power with respect to each statistic on its own. The BOMP burden statistic had more power than BOMP position for the simulations based on a single candidate gene, and vice versa in the simulations with nine candidate genes and 3∶6 causal to non-causal ratio.

We computed average power for single candidate gene case-control studies and multiple candidate gene case-control studies (nine genes, 3∶6 causal to non-causal ratio), with respect to both demographic models, all disease etiologies (Table 1) for single genes, and all combinations of disease etiologies for gene sets (Figure S3). BOMP's hybrid likelihood model had better power than either of its components: the mutation burden and mutation position distribution statistics (Figure 5). The burden statistic had more power in single-gene studies, while the position statistic had more power in the gene set studies.

**Figure 6:**
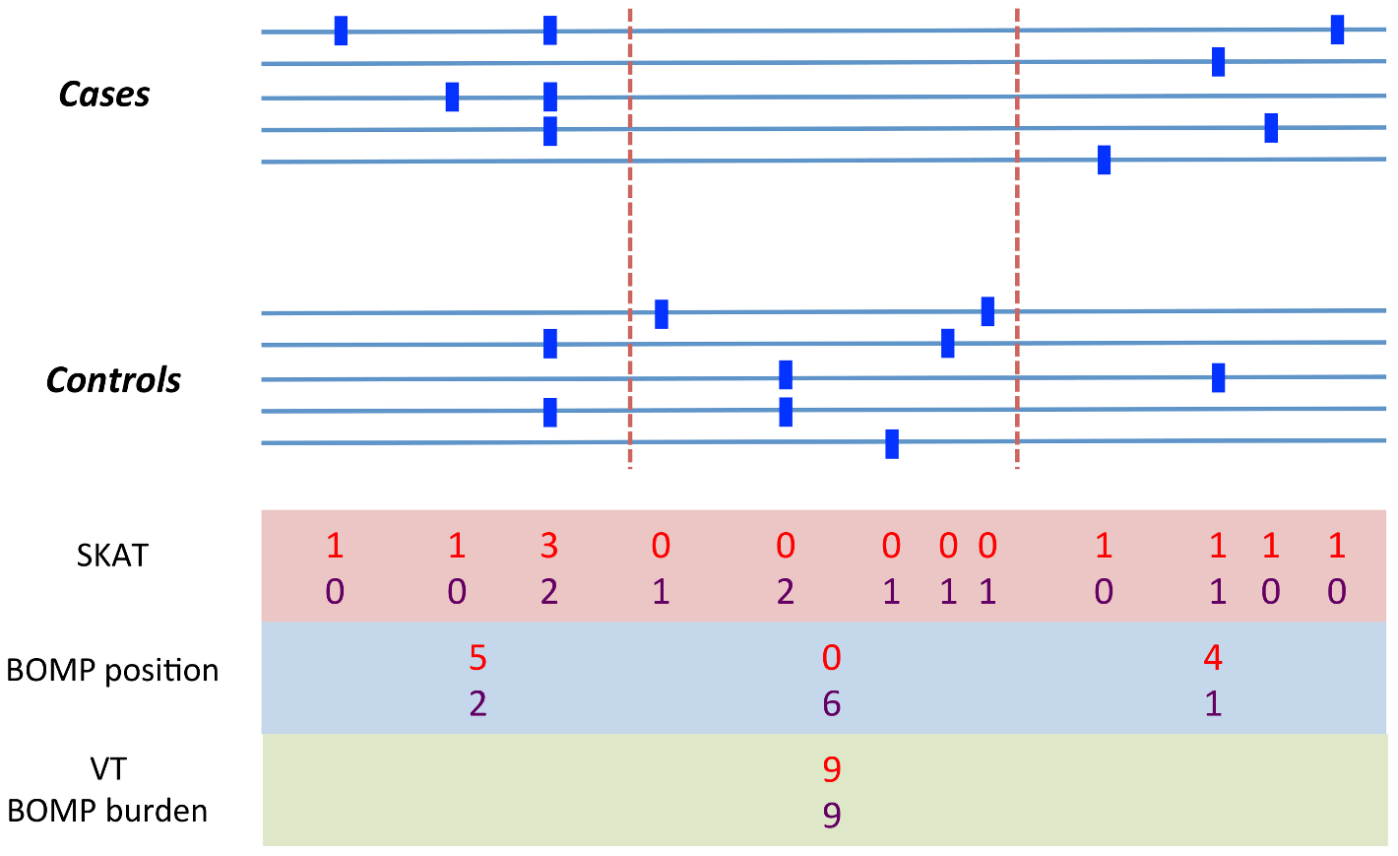
Example variation pattern in which position distribution outperforms burden tests. A toy example of a genomic region containing variants (blue squares) in cases and controls. We assume that the region is important for phenotype. Variant counts in cases (red). Variant counts in controls (purple). Cases and controls each have a total of 9 variants in this region, so Burden statistics (*e.g.*, VT or BOMP burden) will not be able to detect that the region is important for phenotype. BOMP's position distribution statistic collapses variants into short, localized windows (red dashed lines) and detects that the number of variants seen in cases and controls is different within the windows. We note that a method that does not collapse variants, such as SKAT, does not have much power to detect the difference between cases and controls, because at each position the number of variants in cases and controls is similar.

**Figure 7:**
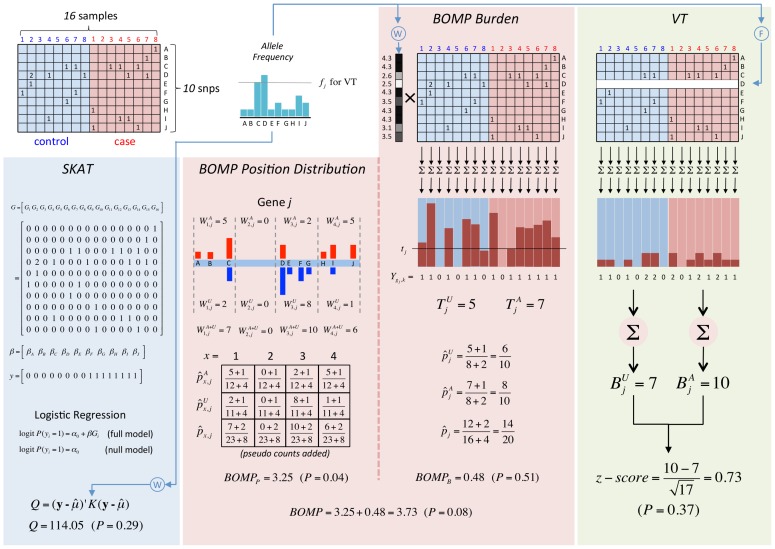
Analytical comparison of SKAT, BOMP, and VT on a toy example. Genotypes of 8 cases and 8 controls at 10 positions. Matrix column colors: controls = light blue, cases = light red. Position distribution bar colors: controls = blue, cases = red. Detailed description is in the section “Toy example with analytical calculations” (Text S1).

In general, mutation burden tests outperform the position distribution statistic when causal variants are rare and are not clustered. The position distribution test outperforms burden tests when the number of rare variants is similar in cases and controls, but where cases and controls differ with respect to the position distribution of the variants. To illustrate this point, we show a case in which burden tests would miss such a difference (Figure 6). In the genomic region shown, cases and controls each have 9 total variants, but an informative window segmentation yields distinct regions in which the number of variants seen in cases and controls is substantially different. The difference between cases and controls is also missed by SKAT, which consider variants one at a time, because at each position the number of variants in cases and controls is similar. (A more detailed example is shown in Figure 7.)

Both collapsing burden and position distribution tests outperform SKAT when causal variants are very rare. Figure S4 shows the power of VT, SKAT, BOMP burden, and BOMP position distribution in 10,000 simulated European-American individuals, using our Rare and Key Region Variant disease etiologies. The European-American populations contain a large fraction of rare variants (Figure S2 shows exact allele frequencies and raw counts). Our simulations of the Rare Variant etiology in this population generate rare variants that are not clustered, and the methods with highest power are VT burden and BOMP burden tests. In Key Region Variant simulations where rare variants are positioned differently in cases and controls, the position distribution statistic has higher power than either of these burden tests.

### Type 1 error

Because we used permutation to compute p-values, type I error should be well controlled.

### Dallas Heart Study

We applied the BOMP hybrid likelihood model to the analysis of data from the Dallas Heart Study (DHS) [17]. Romeo *et al.* explored genetic contributions to plasma triglyceride (TG) levels in 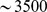 individuals in the DHS, by resequencing the coding regions of several genes, including angiopoietin-like (ANGPTL) family genes. The ANGPTL genes regulate the activity of a key enzyme in TG metabolism, lipoprotein lipase (LPL), *via* post-transcriptional modifications and were jointly associated with low triglyceride levels by [17]. Specifically, *ANGPTL3*, *ANGPTL4*, and *ANGPTL5* were functionally validated as causal genes, playing non-redundant roles and underlying TG levels as a functional group [17]. The ANGPTL gene set has been analyzed in several computational papers and used as a benchmark to compare methods that predict the impact of rare and common variants from sequencing data [10], [12], [13], [20]–[23].

We stratified the DHS samples by ethnicity (Hispanic, non-Hispanic white, non-Hispanic black) and gender. Because BOMP was designed for dichotomous phenotypes, we selected the lower and upper quartiles from each group, by TG level (totaling 1775 individuals, with 897 cases and 878 controls). Sixty mutations in *ANGPTL3*, *ANGPTL4*, and *ANGPTL5* occurred in these individuals.

We computed a P-value for each of the three ANGPTL genes and for the ANGPTL gene set, using BOMP (with and without bioinformatics scores), the burden statistic VT (with and without bioinformatics variant weighting), the overdispersion statistic SKAT, and the mixture-model KBAC statistic (with four parameter settings) (Table 2).

**Table 2:** Dallas Heart Study.

Method	ANGPTL	*ANGPTL3*	*ANGPTL4*	*ANGPTL5*
Hybrid BOMP+VEST	**2.6E-05**	0.09	2.3E-05	0.15
Hybrid BOMP	3.7E-05	0.14	4.3E-05	0.14
VT+VEST	8.3E-05	**0.015**	1.7E-05	0.18
SKAT	1.06E-04	0.068	5.78E-05	0.29
Positional BOMP	1.5E-04	0.4	**1.6E-05**	0.3
KBAC (1D,5P)	2.9E-04	0.031	1.5E-04	0.17
KBAC (2D,5P)	5.5E-04	0.064	3.2E-04	0.31
KBAC (1D,1P)	2.9E-03	0.24	0.033	**0.023**
VT	3.8E-03	0.04	4.56E-03	0.1
KBAC (2D,1P)	5.8E-03	0.47	0.067	0.045
Burden BOMP+VEST	0.006	0.04	0.008	0.09

P-values of association between dichotomized triglyceride levels and variation in three ANGPTL family genes sequenced in Dallas Heart Study. ANGPTL - multiple gene set including *ANGPTL3*, *ANGPTL4*, and *ANGPTL5*. The most significant P-value for each is highlighted in bold. BOMP = combined Burden and Position statistics VT = variable threshold burden test [10] SKAT = sequence kernel association test (linear weighting version) [12], KBAC = Kernel-based adaptive cluster [20] (1D = single direction, 2D = two direction, 1P = rare variants defined as 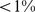 MAF, 5P = rare variants defined as 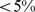 MAF). VEST = BOMP and VT with VEST score variant weighting.

The hybrid BOMP test, with bioinformatics scores and allele frequency variant weighting, had the most significant P-value for the ANGPTL gene set 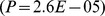, which should be sufficient to detect ANGPTL-phenotype association, using a gene set based analysis in a whole-exome study, after multiple testing correction (Table 2). The hybrid BOMP P-value was more significant than either of its components (the BOMP burden and position distribution scores). This result was consistent with the average behavior of BOMP in our simulation-based analysis of power (Figure 5). However, the two component scores did not yield an improved hybrid score on every gene. For *ANGPTL3*, *ANGPTL4*, and *ANGPTL5*, the hybrid score P-value was not as significant as the P-values of the most significant component score. The burden-based VT score (with bioinformatics score variant weighting) had the most significant P-values for *ANGPTL3* 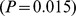; the BOMP position distribution score for *ANGPTL4* 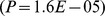, closely followed by VT (with bioinformatics scores) 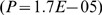, and the overdispersion test SKAT 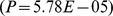. KBAC, with single directional scoring (only deleterious variants counted) and threshold for rare variation set at 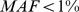, had the most significant P-value for *ANGPTL5* 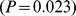.

These results confirm previous reports that the performance of current methods to detect causal variants depends on which genes are selected for benchmarking [6], [14].

### Bipolar case-control study

We then used BOMP to test candidate gene sets in data from an on-going whole exome sequencing study of bipolar disorder. We examined whole-exome sequencing data on the first 191 cases and 107 controls from this study. These samples were sequenced in two rounds, over a two-year period. In the first round, Nimblegen v1.0 arrays were used for exome capture and Illumina GAII platform for next-generation sequencing. In the second round, Nimblegen v2.0 arrays and the Illumina HiSeq2000 platform was used. Only samples with target sequencing coverage of at least 80% at 20× sequencing depth were included for further analysis. Sequence reads from the samples were aligned to the human reference genome sequence database using BWA [24]. Variants were then called after realignment around indels and recalibration of base quality scores with GATK [25] in target regions. Quality control measures for variant calling required coverage of at least 6× depth with a SNP quality score of 30 or higher to eliminate false-positives. Variants were annotated to dbSNP135 and collected in VCF files.

We obtained a collection of pathways for testing from SynaptomeDB [26], a bioinformatics database that we developed to collate available information on genes coding for proteins found in synapses. In SynaptomeDB, a set-based analysis was performed on gene sets from the MSigDB collection [27] to identify gene sets that are enriched for synaptic genes. We extracted twenty gene sets containing at least 100 genes that were most significantly enriched for synaptic genes.

To control for differences in the number of exons targeted by Nimblegen v1.0 and v2.0 (approximately 180,000 vs. 300,000 coding exons) in our analyses with BOMP, we only considered variants in exons present in both Nimblegen kits. We used OverlapSelect from the UCSC Kent source library to identify variants in the shared exons. BOMP P-values and FDR were computed for each of the twenty gene sets selected from SynaptomeDB.

Seven of the gene sets were nominally associated with bipolar disorder (Table 3). The most significant of these, the “MAPK signaling pathway” defined by the KEGG Pathway Database, trended toward significance after correcting for multiple gene sets tested (nominal p-value = 0.0065;FDR = 0.095). This gene set consisted of 267 genes involved in the signaling pathway, including 68 found in neuronal synapses (Discussion).

**Table 3:** BOMP P-values for gene sets in Bipolar case-control study.

Gene Set Name (Source)	BOMP P-value	BOMP Burden P-value	BOMP Position P-value	FDR*	Synaptic Genes	Gene Set Size	Time [m]**
MAPK SIGNALING PATHWAY (KEGG)	0.0065	1.0000	0.0043	0.0949	68	267	87
AXON GUIDANCE (Reactome)	0.0162	0.5847	0.0137	0.0949	71	161	99
NEUROLOGICAL SYSTEM PROCESS (GO)	0.0274	0.2787	0.0273	0.0949	75	377	177
METABOLISM OF PROTEINS (Reactome)	0.0299	0.3437	0.0272	0.0949	96	215	46
NEUROACTIVE LIGAND RECEPTOR INTERACTION (KEGG)	0.0309	0.8490	0.0259	0.0949	21	272	104
HUNTINGTONS DISEASE (KEGG)	0.0312	0.5408	0.0266	0.0949	77	185	51
CALCIUM SIGNALING PATHWAY (KEGG)	0.0332	0.4796	0.0303	0.0949	51	178	85
POST TRANSLATIONAL PROTEIN MODIFICATION (GO)	0.0635	0.4031	0.0620	0.1587	87	462	244
NERVOUS SYSTEM DEVELOPMENT (GO)	0.0744	0.8912	0.0674	0.1654	88	382	157
SIGNALLING BY NGF (Reactome)	0.1290	0.8393	0.1139	0.2317	71	215	103
GNRH SIGNALING PATHWAY (KEGG)	0.1313	0.7485	0.1109	0.2317	34	101	43
OXIDATIVE PHOSPHORYLATION (KEGG)	0.1390	0.4222	0.1263	0.2317	66	135	21
ALZHEIMERS DISEASE (KEGG)	0.2164	0.7012	0.1904	0.3151	76	169	46
INTRACELLULAR SIGNALING CASCADE (GO)	0.2267	0.7489	0.2174	0.3151	120	648	256
NEUROTROPHIN SIGNALING PATHWAY (KEGG)	0.2363	0.7995	0.2031	0.3151	45	126	44
CHEMOKINE SIGNALING PATHWAY (KEGG)	0.2667	0.6638	0.2464	0.3272	50	190	62
WNT SIGNALING PATHWAY (KEGG)	0.2781	0.7009	0.2519	0.3272	38	151	56
REGULATION OF GENE EXPRESSION IN BETA CELLS (Reactome)	0.3401	0.0877	0.4571	0.3779	60	101	10
MITOCHONDRION (GO)	0.4315	0.9727	0.4047	0.4542	117	339	104
PARKINSONS DISEASE (KEGG)	0.7893	0.3087	0.8111	0.7893	62	133	22

The gene sets were selected for testing because they contained 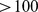 genes and were the most significantly enriched by synaptic genes [26]. Seven of the genes sets were nominally associated with bipolar disorder (P
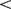
0.05) and have FDR
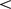
0.1.

* FDR computed with the Benjamini-Hochberg algorithm [45].

** Wall-clock time in minutes.

To check for possible systematic bias in our Bipolar analysis *e.g.*, effects of population substructure, we compared the observed distribution of BOMP P-values with that expected under the null hypothesis of no association. The resulting Q-Q plot (Figure S5) is not heavily skewed or heavy-tailed. We also checked for possible bias resulting from the version changes in exome capture and change from Illumina GAII to HiSeq2000 sequencing platforms between rounds one and two. We performed PCA analysis with EISENSTRAT [28], which revealed no significant differences in the nature or frequency of variants identified in the two rounds (Figure S6).

### Computational time

In our simulations, analysis of an average-sized gene (500 codons), using 100,000 permutations, required wall-clock times of 32 s, 57.8 s, 1 m23 s, and 3 m4.2 s for case-control study sizes of 200, 1000, 2000, or 5000 (African American demographic model) and 22.8 s, 1 m7.6 s, 1 m25.2 s, and 3 m9.6 s for European American demographic model. For our gene set analyses with real data from the bipolar case-control study (298 individuals), using 100,000 permutations to compute P-values, BOMP computation time ranged from 10 m for a gene set with 101 genes to 4 h16 m for a gene set with 648 genes (Table 3). All computations were done on a machine with four dual-core 2.6 GHz AMD Processors and 16 Gb memory.

## Discussion

In this work, we introduce and explore the power of a new hybrid likelihood model BOMP to detect causal variants underlying dichotomous disease phenotypes. We compared its power with that of several leading methods designed to detect causal variation in whole-exome case-control studies. We performed simulated case-control studies, using a variety of sizes, demographic models, and disease etiologies. The hybrid BOMP model had good power compared to several popular methods (Figure 1, Figure 2, Figure 3). Its strengths were most apparent when we tested gene sets for association with phenotype, when protective or common variants were present, and when a phenotype was associated with variants that cluster in key regions on a gene.

Because no current variant collapsing methods have been shown to be best for every disease etiology [6], we were particularly interested in how the performance of BOMP and other methods varied by etiology. Our case-control study simulations were designed to represent eight etiologies (Table 1). Etiologies were defined by properties of their causal variants, based on range of minor allele frequencies, selection coefficients, position distribution, and mean effect sizes. Some etiologies include protective variants, while others include only deleterious variants. For all of our etiologies, only coding, non-silent variants were considered to have an impact. Importantly, our etiologies are defined in a probabilistic way since we allow for a randomly selected fraction of variants which meet the causality criteria for an etiology to have no effect (Methods). As a result, in the rare variant etiology, all causal variants are rare, but not all rare variants are causal, *etc.*. Method performance also varied by the population demographic models (European-American exponential growth and African-American simple bottleneck) used in our simulations. We provide details of how the performance of all tested methods was affected by disease etiology and demographic model in Text S1.

### Gene set simulations

We considered the possibility that differences between cases and controls might be detected with respect to a gene set, rather than a single gene [29]. We tested the methods, using gene sets of different sizes (Figure 2), different combinations of disease etiologies (Figure 3, Figure S3), and different fractions of genes containing causal variants (Figure 2). Of the tested methods, burden statistics and KBAC were the least effective at detecting gene sets containing causal variants. SKAT and the BOMP position distribution statistic were the most effective.

Biologically, we don't expect that every gene in a real gene set will contain causal variants. Thus our simulated gene sets were designed to contain a mix of genes with causal variants and those without. The burden tests (VT and BOMP burden) were not able to effectively capture the difference between the two and lost power as the number of non-causal variants in the simulations increased (Figure 2). The genotype vectors computed by KBAC become larger and more heterogeneous when applied to a gene set, rather than a single gene. Thus, the KBAC strategy of leveraging the number of shared genotype vectors among cases and/or controls is less effective when applied to gene sets than to single genes. The BOMP hybrid likelihood statistic (with strong contributions from the BOMP position distribution statistic), and SKAT were the most powerful when applied to gene sets, rather than single genes. We attribute this result to the increase in the number of significant localized units in a gene set that contains more than one causal gene.

We found in our simulations that a case-control study size of 1000 individuals (500 cases, 500 controls) BOMP was sufficiently powered to detect causal variants in situations when a good candidate gene set (of approximately 25 genes) was known. However, if the ratio of causal to non-causal genes in the selected gene set was low (1∶3) and/or the individuals in the study carried a high proportion of rare (MAF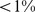) or singleton variants, the power of the BOMP hybrid model, like all the methods examined, was diminished. This result highlights the importance of very high quality gene sets derived either from disease experts or improved bioinformatics tools for success in real data analysis.

### Dallas Heart Study benchmark set

When we applied BOMP, VT, SKAT, and KBAC to an empirical dataset, each method displayed both strengths and weaknesses. While the dataset is small, it is interesting to note that P-values of association between variant ANGPTL family genes and dichotomized serum triglyceride levels from the Dallas Heart Study were most signicant for the BOMP hybrid model, when the genes were considered together as a gene set. However, the burden statistic VT had the most signicant P-value for *ANGPTL3*, and the KBAC P-value was the most significant for *ANGPTL5* (specically with single directional scoring and threshold for rare variation set at MAF 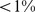). For *ANGPTL4*, the most signicant P-values were from the BOMP position distribution score, VT, and SKAT. Each of these genes had a different pattern of variation frequencies in cases and controls, which presented advantages and obstacles for each method. For example, *ANGPTL3* had a high frequency variant (M259T) that occurred more often in cases than controls and many “noisy” rare/singleton variants that occurred either in cases or controls. VT took advantage of the signal in M259T because its threshold adapted to maximize the burden increase in cases versus controls, and thus M259T was included in its burden calculation. BOMPs burden statistic did not give as much importance to M259T, because it downweights high frequency variants. KBAC included M259T only when its allele frequency threshold parameter was set to 5% but was penalized when it was set to 1%.

BOMP is not designed to be adjusted for additional covariates, which are often available in disease studies. For example, it is not designed to explicitly deal with different ancestries in a structured population. However, if the true population structure is known and the number of subpopulations is not too large, we can run analyses with stratification to get around this problem, as we (and the authors of the VT and SKAT papers) did for ANGPTL family genes in the Dallas Heart Study [10], [12]. Using this strategy, one begins with a quantitative trait (serum triglyceride levels), stratifies individuals into groups, then identifies extreme phenotype individuals from each group. Cases are then those individuals from all groups at one extreme and controls are those individuals from all groups at the other extreme. An alternative strategy is to permute case-control labels only within each group to generate a correct null distribution.

Incorporating bioinformatics scoring of variants (by VEST) yielded improved P-values for both BOMP and VT on the Dallas Heart Study data. While it has been suggested that bioinformatics misclassification of variants might be more of a liability than a benefit, our results (albeit on a small gene set) suggest the opposite. Functional classification of variants in both coding and non-coding regions of the genome is an active research area in bioinformatics, and as methods improve, it is likely that they will increasingly contribute to statistical analysis of causal variation.

### Bipolar whole-exome sequencing, case-control study

Finally, we applied BOMP (with VEST scoring) to test candidate gene sets in data from an on-going whole-exome study of bipolar disorder. The top gene set was the “MAPK signaling pathway” defined from the KEGG Pathway Database (map04010). This is a highly conserved pathway that is centrally involved in cell proliferation, differentiation and migration. In the nervous system, it is at the nexus of multiple neuronal signaling cascades thought to mediate certain forms of synaptic plasticity [30]. Interestingly, the pathway has been implicated before in the etiopathogenesis of bipolar disorder [18] and a number of studies have shown that two most commonly used anti-manic agents, lithium and valproate, activate the MAPK signaling cascade, which may in part be responsible for their therapeutic effects [31]. One of the key genes in this pathway, DUSP6, a cytoplasmic phosphatase that plays an active role in MAPK signaling by regulating the intensity and duration of MAPK activity and by helping to shuttle MAPK between the cytoplasm and nucleus, has been associated with bipolar disorder [32], [33]. There are several other genes of considerable interest in this gene set, as they have been implicated previously in the genetics of bipolar disorder. For example, this pathway also contains CACNA1C and other members of the family of voltage gated calcium ion channels. CACNA1C was recently implicated as one of the most significant genes in a mega-analysis of genome-wide association studies (GWAS) of bipolar disorder carried out by the Psychiatric GWAS Consortium (PGC) [34]. This gene set also contains the phospholipase A2 family of genes. Phospholipases mediate the release of arachidonic acid from membrane phospholipids. Arachidonic acids are involved in brain signaling processes that mediate a number of effects, and there is growing evidence that the arachidonic acid signaling cascade may be a common target of multiple diverse mood stabilizers [35].

Complex diseases are expected to have considerable genetic heterogeneity *i.e.*, they may be the consequence of alterations in hundreds of potentially causal genes, and affected individuals may have causal and/or protective variants in different subsets of these genes. The simulations done in this work reflect this heterogeneity (example in Figure S7), and we still retain good power.

Bayesian extensions of our work could include prior knowledge about the probability that a functional group of interest is associated with the phenotype. For example, if the functional group is a gene, prior evidence could come from expert knowledge based on previous functional and/or case-control studies. By combining the log likelihood ratio (Equation 1) with a log prior ratio, we could estimate the log ratio of the full posterior distributions.

In summary, we have developed a new method for identifying causal variants in high-throughput sequencing data from case-control studies. It is shown to have good power relative to other leading methods and can be flexibly used in a variety of realistic scenarios. The genetic architecture of most common human diseases is likely complex, involving variants with a wide spectrum of frequencies from rare to common and contributing to disease through a number of inter-related pathways. The emergence of whole-exome and genome sequencing studies promises to accelerate our ability to interrogate the genetic architecture of these disease. However, a major challenge remains how to make sense of the enormous amounts of data generated by such studies. Our new method provides another useful tool in a growing toolbox for analyzing the data from such studies.

## Methods

BOMP (Burden Or Mutation Position statistics), the hybrid likelihood model proposed here, consists of two likelihood ratio tests (mutation burden and mutation position distribution statistics) with the same general form,
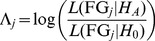
(1)and tests the evidence for the alternative hypothesis 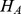 that a functional group (FG) of interest is associated with a disease phenotype, compared to the null hypothesis 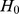 that they are not associated. Higher values of 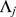 indicate stronger association between unit 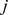 and the disease phenotype. In this work, the functional groups of interest are either single genes or sets of multiple genes.

### BOMP mutation burden statistic

The first likelihood ratio test is based on comparing mutation burden in cases and controls. Each individual is represented with a Bernoulli random variable, which is 1 if the individual's burden exceeds a burden threshold, and 0 otherwise. To model the likelihood, we assume that individual burden status is independent and identically distributed (IID). The ratio compares an alternative hypothesis that the probability of exceeding the burden threshold is higher in cases than in controls and the null hypothesis (that probabilities are equal or lower in cases than in controls). Biologically, the IID assumption is not necessarily true. We control for such violations by assessing the statistical significance of the likelihood ratio by permuting case and control labels (Figure S8).

#### Individual burden

For individual 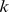 the gene burden of 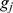 is
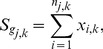
(2)where 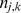 is the number of variants carried by individual 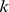 in gene 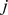 and 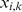 is the genotype of variant 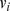.

#### Individual burden thresholds

A binary variable is used to label individuals whose mutation burden in a gene of interest exceeds a critical threshold. If the burden of gene 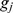 in individual 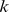 is greater than or equal to the threshold 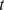, then it is considered to be *disease phenotype associated* for that individual and 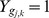 (0 otherwise). Because genes are heterogeneous in size, functional importance, mutation rate, and tolerance to variation, each gene may have a different value of 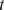. For each gene 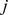, this cutoff 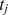 is computed by iterating over all cut-offs and selecting the one that maximizes its mutation burden statistic (Equation 4).

#### Aggregated burdens

**Figure 8:**
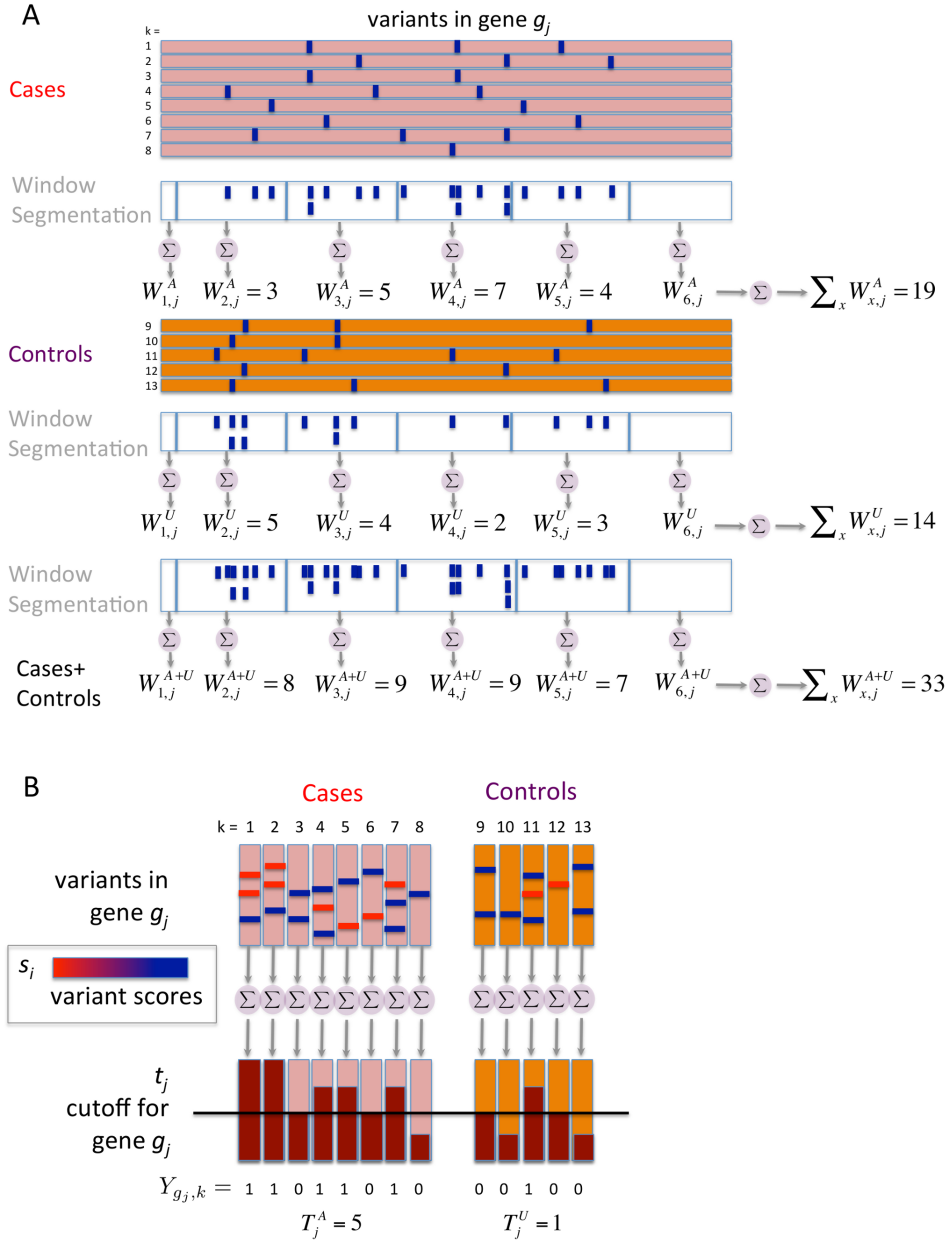
Components of BOMP Hybrid Likelihood Model compared. A. Mutation burden statistic. The Mutation burden statistic uses the aggregated burden for cases, 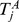, and controls 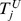. B. Mutation position distribution statistic. Aggregated window mutation counts are calculated for cases, 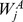, controls, 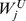, and cases and controls combined, 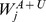, across 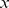 windows.

The 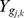 values are then aggregated by summing over cases and controls:
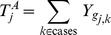


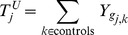
The maximum likelihood estimate of the probability that the mutation burden of gene 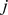 exceeds the threshold in cases is then 
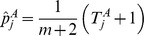
, the estimate for controls is 
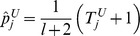
 and the estimate for both cases and controls is 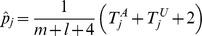, where 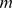 is the number of cases and 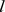 the number of controls. The probability estimates 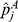, 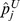, and 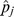 are used as the parameters of three Bernoulli distributions (one for cases, one for controls, and one for cases and controls together). Pseudocounts are added to avoid zero counts. The aggregated burden calculation (without pseudocounts) is illustrated in Figure 8 (and Figure 7).

#### Burden likelihood ratio statistic

For a gene 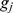 the mutation burden statistic is defined as a ratio of Bernoulli likelihoods:
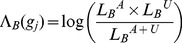
(3)

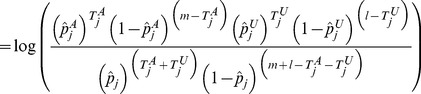
(4)where 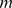 is the number of cases, 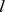 the number of controls; 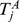 is the number of cases whose mutation burden in 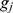 exceeds an optimized threshold (Individual Burden Thresholds); 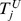 is the number of controls exceeding the threshold; 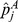 is the maximum likelihood estimate of the probability that the burden of gene 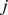 exceeds the threshold in cases, 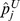 is the estimate that gene 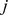 exceeds the threshold in controls, 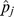 is the estimate that gene 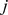 exceeds the threshold in both cases and controls. First, we consider only genes with higher burden in the cases, for which 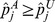. Next, for the remaining genes, we modify Equation 4,
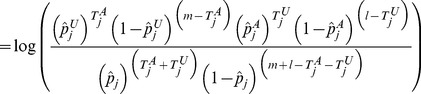
(5)


Thus genes with higher burdens in cases than controls get a high value and those with higher burdens in controls than cases get a low value.

It follows that under 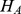, the number of cases in which the burden of gene 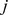 exceeds the threshold will be larger than in controls, and that under 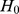, they will not be different.

If a gene set rather than a single gene is used as the functional group, the burden is aggregated across all genes in the set, and the procedure is otherwise identical.

### BOMP mutation position distribution statistic

The second likelihood ratio test is based on comparing the position distribution of mutations in cases and controls. The codons of a gene are partitioned into windows and mutation count (burden score) is computed for each window in cases only, controls only, and in cases and controls together. To model the likelihood, each window mutation count is considered to be a random variable in a multinomial distribution. If the partition contains 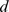 windows, there are 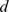 possible outcomes for each mutation. There are also 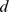 multinomial parameters for the partition.

#### Window mutation counts

Let the window mutation counts in the multinomial distributions be 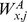, 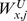, and 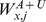 (cases only, controls only, and in cases and controls together)
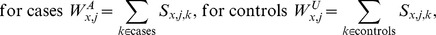


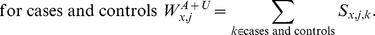
where 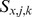 (computed as in Equation 2) is the score for individual 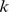 in window 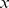.

The maximum likelihood estimate of the multinomial parameters (including pseudocounts) is then
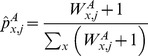
(6)

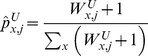
(7)

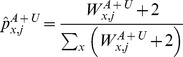
(8)


#### Position distribution likelihood ratio statistic

For a gene 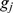, the statistic is defined as a ratio of multinomial likelihoods:
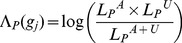
(9)where
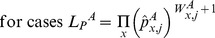
(10)

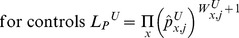
(11)

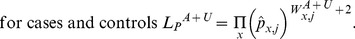
(12)


It follows that under 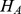, the likelihood for cases will be different than for controls, and that under 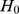, they are not different. In contrast to the mutation burden statistic, there is no directionality in the mutation position distribution statistic, because 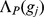 will be large when either 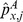 or 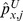 is large.

A toy example of aggregated window mutation count calculation is illustrated in Figure 8 (and Figure 7).

#### Windows and sequence segmentations

Each gene has many possible window partitions, and we don't know in advance which is the most informative for the position distribution statistic. One way to create candidate window partitions (*i.e.*, sequence segmentations) for a gene of length 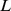 is to select a window size 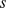 and a series of possible offsets, based on a selected shift increment 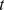. Each offset generates a new segmentation (Figure S9). For example, if the window size is 8 and the shift increment is 1, the first offset begins at the first position of the gene and generates a segmentation of 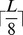 windows. The second offset will begin at position 2 of the gene and generate a new segmentation of 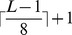 windows, *etc.* In this work we used four combinations of window size 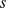 and shift increment 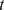: (8,1), (16,2), (32,4) and (64,8), yielding 32 candidate segmentations for a gene. These choices were not optimized and can be adjusted, according to user preference and/or prior knowledge. The best segmentation is selected by computing the likelihood ratio 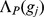 (Equation 9) for each segmentation and picking the segmentation with the largest 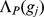. Alternatively, this likelihood ratio can be modified by computing 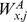, 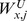, and 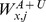 with respect to total positions mutated, rather than total number of mutations.

For the position distribution statistic, if a gene set rather than a single gene is used as a functional group, the best window segmentation is computed for each gene, and the calculation of the position distribution statistic is otherwise identical.

The mutation burden and mutation position burden statistics are combined into a single log likelihood ratio,
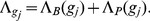
(13)


### Controlling false positives through permutation

P-values for each 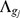 are computed with a null distribution, generated by repeated permutation of case and control labels. All parameters of 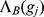 and 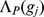, including the maximum likelihood burden threshold and segmentation pattern, are calculated initially for empirical data and then re-calculated for each iteration of the permutation. Thus, 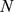 iterations yields 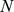 null 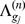 where 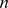 ranges from 1 to 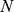 (Figure S8, Figure S10). The permutation controls for confounding effects, such as properties that characterize a particular gene or region of interest (*i.e.*, nucleotide diversity, GC content, and recombination rate), which are the same when used to estimate 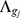 and each null 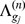.

After 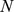 iterations (*e.g.*, 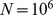 )
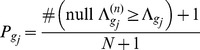
(14)While 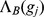 and 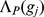 are not independent, using the permutation test yields an accurate P-value estimate, because any dependencies in 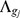 are reproduced in each 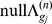.

### Extensions to the basic method

#### Genetic models

Either *dominant* or *additive* genetic models can be specified. Under the dominant genetic model, both homozygous and heterozygous variants have 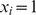; under the additive model, homozygous variants have 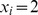 and heterozygous variants 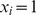. For all experiments in this work, additive models were used.

#### Variant scores

The Individual Burden can be modified by incorporation of score coefficients so that 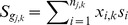. The score for a variant 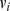 can be either a bioinformatics-based score 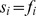, an allele-frequency-based score 
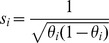
, following [9], [10]; or the product of both 
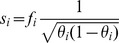
. Next we explain how these scores are calculated. In this work, variant scores were used only in the burden statistic. The allele-frequency-based score was used for all simulations and the product of allele-frequency and bioinformatics scores was used on all empirical data.

#### Bioinformatics scores

Each nonsilent variant 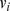 can be assigned a score 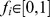 to represent its contribution to a disease phenotype of interest, where 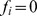 indicates no contribution and 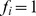 indicates a strong contribution. These scores are estimated with *Variant Effect Scoring Tool* (VEST). Variants causing nonsense, nonstop or frameshift alterations to a gene's protein product receive 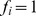. Variants causing missense alterations are scored with a Random Forest classifier [36], [37]; the score is the fraction of decision trees in the forest that classified the variant as deleterious. Alternatively, other bioinformatics methods that score missense variants can be used to generate 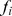 values, if scaled to range from 0 to 1.

The Variant Effect Scoring Tool is a Random Forest classifier, trained with the CHASM software suite's Classifier Pack and SNVBox [38]. The Forest contains 1000 decision trees. The positive class of 45,000+ missense variants is taken from the Human Gene Mutation Database (HGMD) [39]. The negative class of 45,000+ missense variants is randomly selected from variants validated by the 1000genomes project [40] in the SNP135 table of the UCSC Genome Browser database [41]. Each missense variant is represented by 86 features in SNVBox, including conservation scores, amino acid residue substitution scores, UniProtKB annotations [42], and predicted local protein structure.

#### Allele-frequency-based scores

For each 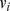, we estimate its mean population allele frequency 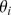 as follows:
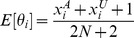
(15)where 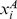 and 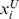 are allele counts of variant 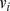 in cases and controls, respectively; 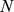 is the number of individuals in both cases and controls; and the constants are pseudocounts from a beta prior.

### Simulation framework

Simulated case-control studies are generated using two demographic growth models, eight disease etiologies, and a stochastic model of genotype-phenotype association.

#### Generating genomic populations

The general Wright-Fisher model/forward population genetic simulation tool SFS_code [43] is used to generate 100 effective genomic populations and to sample 1,000,000 haplotypes per population. As in [13], the simulated haplotypes in each population are randomly paired to generate 500,000 diploid individuals. Two demographic growth structures are used: an exponential growth model fitted to deep resequencing data from European Americans [19] and a simple bottleneck model fitted to whole-genome polymorphism data from African-Americans [44]. For the exponential growth demographic model, *distribution of fitness effects* of new mutations (DFE) is modeled with a two-component gamma mixture (similar to [19]). For the simple bottleneck model, DFE was modeled as described in [44] (Text S1:Demographic Models). Following [19], the mutation rate is set to 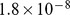 per generation for all simulations.

#### Generating phenotypic traits for individuals with a single causal gene

The individuals in a population are then associated with a quantitative phenotypic trait, which we assume drives a disease, so that individuals with high values of the trait will have the disease and those with low values of the trait will not. Eight possible disease etiologies are considered (Table 1). Each etiology is defined by properties of its causal variants. Variants can be rare, low frequency, or common. They can occur only in key functional regions. They can have small or large effects (value of 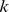 in Equation 17). Protective modifier variants may or may not be present. In this work, only coding, non-synonymous variants are considered as causal or protective for all etiologies. (However, disease etiologies that consider silent variants, which impact gene regulation, could also be defined.) For etiologies with key region variants, we used haplotypes that contained multiple coding segments (100 segments, each 30 bases long). Otherwise haplotypes contained a single coding segment of 1500 bases.

To generate the phenotypic traits for the genomic populations, we select a disease etiology and a population that contains variants meeting the criteria for causality in that etiology (Table 1).

Next the quantitative disease trait 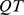 is generated for each individual in the population, using an approach based on [19]. Trait values are drawn from Gaussian distributions, such that individuals with no causal variants have
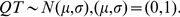
(16)Individuals with 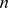 causal variants have
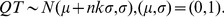
(17)where 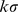 is the mean shift in trait value (the shift per variant) for an individual.

To match the expected effect size of significant common and rare variants in GWAS, effect sizes of 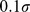 for common variants (Text S1: Effect size of common variants) and 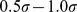 for rare variants are used. The strongest effects are 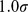 for rare variants in Key Regions. We assume that these variants occur at functionally important positions and that in a gene of interest, they are unlikely to occur more than once in a single individual. Our choice of effect size is somewhat larger than that used by Kryukov *et al.* [19], who use 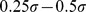 for rare variants. To account for heterogeneity within a particular disease etiology, effect size can be 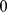 for a designated fraction of causal variants.

#### Case-control study generation

At this stage, each population consists of genomic individuals, each with a real-valued quantitative trait. To construct the case-control studies, an extreme phenotype model is used. Disease prevalence is set at 1%, *i.e.*, the 1% of individuals in the selected population with the highest values of 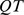 are considered *affected* and the 25% of individuals with the lowest values of 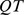 *unaffected*.

Case-control studies are generated by sampling without replacement from affected and unaffected groups in a population. Individuals with intermediate phenotype values are not included in case-control studies. The random process used to generate 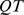 (Generating phenotypic traits for individuals with a single causal gene) ensures varied penetrance and phenocopy rates in each case-control study, *e.g.*, some individuals carrying deleterious variants are not affected, while some with no deleterious variants are affected.

#### Null case-control study

A null case-control sample is also generated, with no disease etiology, in which the phenotypic trait is drawn from a standard normal distribution for every individual in the sample.

#### Generalization to multiple genes

For a scenario in which the functional group of interest is a *gene set e.g.*, involved in a pathway or biological process, we construct a new population of 500,000 individuals, in which each individual has multiple genes. This population is created by sampling genes from the diploid gene populations generated previously (Generating genomic populations). Next, we specify a gene set size and fraction of genes in the gene set that contain causal variants. A disease etiology is then randomly selected for each gene that contains causal variants. Finally, the phenotypic trait for each individual in the population is generated using Equations 16 and 17. Gene set case-control studies were generated with the same protocol as for single causal genes.

## Supporting Information

Figure S1Power estimates for BOMP, VT, SKAT, KBAC for case-control study with 10,000 individuals. (KBAC1P = minor allele frequency defined as 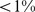, KBAC5P = minor allele frequency defined as 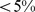). Each column represents power estimates for each method, based on 250 simulated case-control studies. All case-control studies had 10,000 genomic individuals, each with a single gene. AA = the case-control studies were drawn from gene populations generated with an African-American simple bottleneck demographic model. EA = the case-control studies were drawn from gene populations generated with a European-American exponential growth demographic model. The eight variant causality (disease etiology) models are defined in Table 1. Since the European-American demographic model does not account for common or protective variants, etiologies involving common or protective variants were only considered for the African-American demographic model.(PDF)

Figure S2Distributions of allele frequencies and raw allele counts in simulated European-American and African-American populations. The European-American population consists almost entirely of rare variants, while the African-American population contains a wider range of rare, low-frequency, and common variants. Percentage of variants with allele frequencies and raw allele counts in the designated ranges are shown. Because 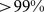 of European-American allele frequencies are 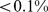, we include a blow-up of frequencies 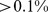, which range from 0.05% to 0.2%. Demographic models shown in Figure S11.(PDF)

Figure S3 Nine multinomial distributions used to construct sets of multiple candidate genes for case-control studies. Each multinomial distribution is named for its dominant disease etiology.
(PDF)

Figure S4 Power of position distribution statistics compared to burden methods and SKAT. Burden tests outperform the position distribution statistic when causal variants are rare and are not clustered, as in our simulations of Rare Variant disease etiology and European-American demographic. The position distribution test outperforms burden tests when the number of rare variants is similar in cases and controls, but where cases and controls differ with respect to the position distribution of the variants, as in simulations of Key Region Variant disease etiology and European-American demographic. Both collapsing burden and position distribution tests outperform SKAT when causal variants are very rare. RareEA = rare variant disease etiology (Table 1) and European-American demographic model. KeyRegionEA = key region variant disease etiology (Table 1) and European-American demographic model. Power shown on Y-axis. Simulations with 10,000 samples are shown.
(PDF)

Figure S5 Q-Q plot of BOMP P-values for all genes in the Bipolar case-control study. The plot shows no evidence of heavy skew or heavy tails, indicating that there is no systematic bias in our analysis. Empirical P-values are below the line because the BOMP statistic is not continuous for genes with few variants in only a few samples, leading to conservative P-values.
(PNG)

Figure S6 PCA plot showing the overlap of bipolar samples and controls sequenced during rounds 1 and 2. Plot obtained using EIGENSTRAT [28], where the first and second components of the model (PC1 and PC2, respectively) are shown. The analysis does not show significant differences in the nature or frequency of variants identified in the two rounds. Round 1 = Nimblegen v1.0 and Illumina GAII, Round 2 = Nimblegen v2.0 and Illumina HiSeq2000.
(PNG)

Figure S7 Example of how our simulations capture genetic heterogeneity in complex disease. Each horizontal grid line represents a genomic individual. (Cases and controls shown separately.) Each vertical gridline represents a gene. Causal variants (both deleterious and protective) are shown as triangles. Different case individuals have different patterns of causal variants and the allele frequencies of the variants range from rare (1 allele) to common (190 alleles). Causal variants are also observed in the control individuals. Type = Downward pointing triangles are deleterious variants, upward pointing triangles are protective variants. (200 genomic individuals from African-American demographic model are shown).
(PDF)

Figure S8Flow chart for calculation of mutation burden statistic. The statistic is first calculated from empirical data (by following the blue and black arrows). The null mutation burden statistics are calculated by following the red and black arrows. Key steps in the calculation are: computing the burden for each individual; sorting the individual burdens into a ranked list, where 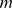 denotes list rank; selecting a candidate burden threshold; computing maximum likelihood estimates of Bernoulli parameters at this threshold; computing the LLR (log-likelihood ratio) with these parameters. Candidate burden thresholds are iteratively tested and the threshold yielding the largest LLR is returned. To generate the null distribution, case-control labels are repeatedly permuted and the key steps are followed to compute one point in the null. The permuted null distribution is estimated by repeating steps 1–4 10,000 times (or user-adjusted value) and used to compute the p-value for the statistic.(PDF)

Figure S9Window and sequence segmentations. The mutation position distribution statistic requires a segmentation for a sequence of interest (*e.g.*, a gene). We generate candidate segmentations by selecting a window size 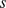 and allowing a series of possible offsets, based on a selected shift increment 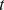. In this example, we illustrate the eight possible window segmentations of a gene with 24 codons (represented by rectangles), using a window size of 8 and a shift increment of 1.(PDF)

Figure S10Flow chart for calculation of position distribution statistic. The statistic is first calculated from empirical data (by following the blue and black arrows). The null position distribution statistics are calculated by following the red and black arrows. Key steps in the calculation are: choice of (user-selected) window sizes 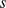 and shift increment 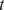 to generate a set of gene segmentations (Each unique segmentation is defined by 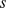, 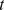, and 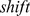, which represents the current segmentation offset); estimation of 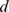 parameters for each of three multinomial distributions – cases, controls, and cases and controls together – (for a segmentation with 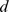 windows); computation of log likelihood ratio; finding the largest log likelihood ratio for all segmentations. To generate the null distribution, case-control labels are repeatedly permuted and the key steps are followed to compute one point in the null. The permuted null distribution is estimated by repeating steps 1–4 10,000 times (or user-adjusted value) and used to compute the p-value for the statistic.(PDF)

Figure S11 Demographic models of European-American and African-American populations. The models were fit to European-American [19] and African-American sequencing data [44]
(PDF)

Table S1 BOMP, VT, and SKAT comparison. Approaches to variant collapsing, variant importance, and choice of statistical framework define differences and similarities among BOMP, VT, and SKAT. A. Variant collapsing strategies. VT and BOMP burden both collapse variants across a genomic region. SKAT does not do collapsing and considers variants one at a time. BOMP position distribution collapses variants across local windows over a genomic region. These different collapsing strategies are illustrated in a toy example in Figure 8 of our main manuscript. B. Variant importance. All methods assume that some variants are more important than others. This idea is implemented by either filtering and/or weighting variants. C. Statistical framework. VT and BOMP burden both use a summary statistic to compare burden in cases and controls and assess the statistical significance of the statistic with permutation. For VT, individual burdens are summed over the case group and over the control group and summarized by the difference in Z-score between the two groups. For BOMP burden, individual burden is dichotomized for each sample, by selecting a burden threshold. The probability of exceeding the burden threshold for cases and for controls is estimated. The difference between the two is compared with a log-likelihood ratio. For SKAT, phenotypic labels (case or control) are directly regressed for each variant. The coefficient for each variant is tested by comparing it to 0, with a variance-component score test. Statistical significance is calculated analytically. For BOMP position distribution, the difference of distributions of variants over local windows between cases and controls is modeled by multinomial likelihood and then summarized by a log-likelihood ratio.
(PDF)

Text S1 Supplementary Material.
(PDF)
