# Supplementary figures and images for: A Hybrid Likelihood Model for Sequence-Based Disease Association Studies
Source: PLoS Genet. 2013 Jan 24;9(1):e1003224. doi: 10.1371/journal.pgen.1003224 (PMC3554549; doi:10.1371/journal.pgen.1003224)

## Case-control study size = 10000

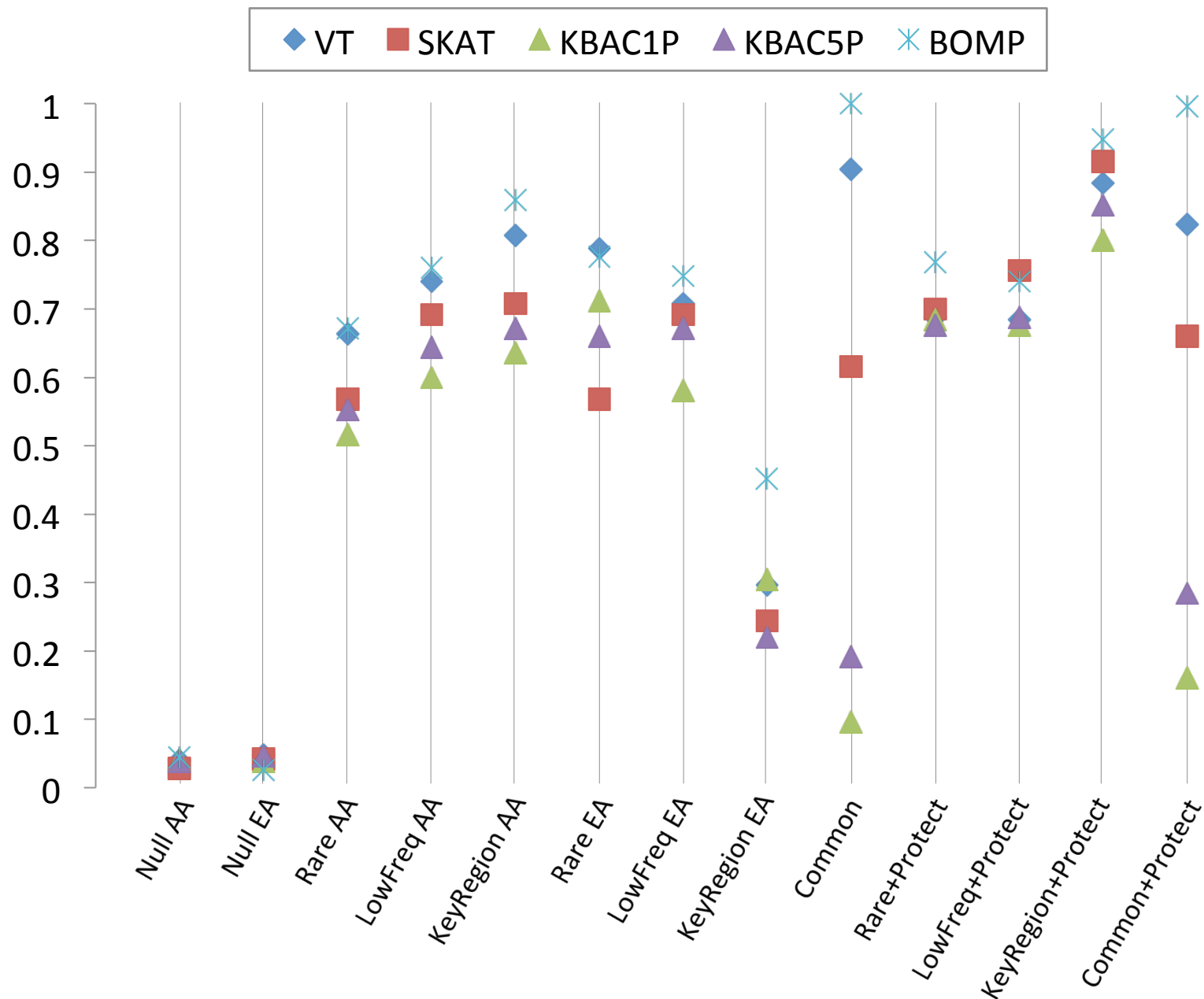

Supplement: Figure S1 — Power estimates for BOMP, VT, SKAT, KBAC for case-control study with 10,000 individuals. (KBAC1P = minor allele frequency defined as , KBAC5P = minor allele frequency defined as ). Each column represents power estimates for each method, based on 250 simulated case-control studies. All case-control studies had 10,000 genomic individuals, each with a single gene. AA = the case-control studies were drawn from gene populations generated with an African-American simple bottleneck demographic model. EA = the case-control studies were drawn from gene populations generated with a European-American exponential growth demographic model. The eight variant causality (disease etiology) models are defined in Table 1. Since the European-American demographic model does not account for common or protective variants, etiologies involving common or protective variants were only considered for the African-American demographic model. (PDF) [file pgen.1003224.s001.pdf]

### AA simple bottleneck

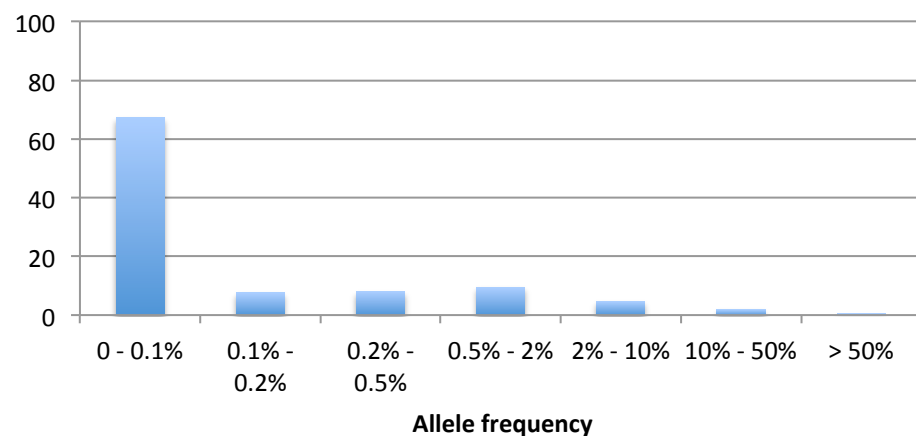

### EA exponential growth

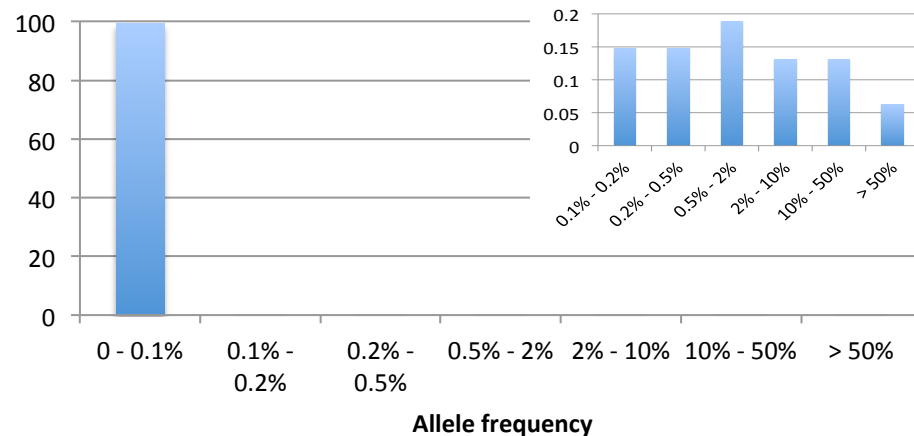

### AA simple bottleneck

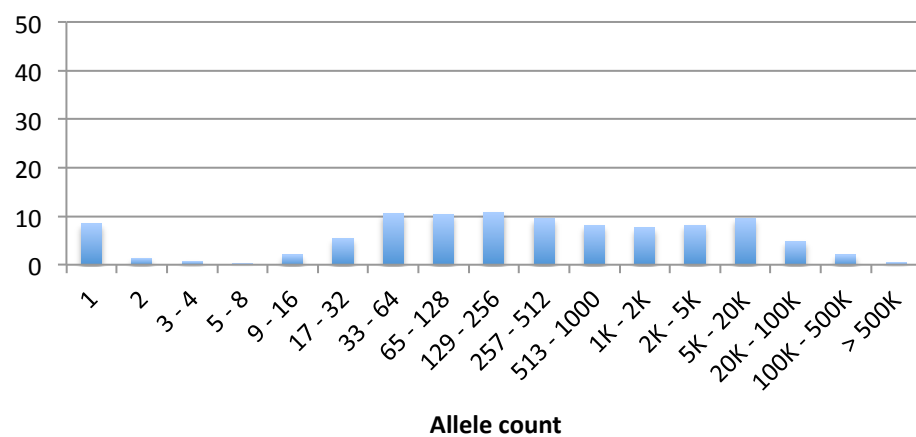

### EA exponential growth

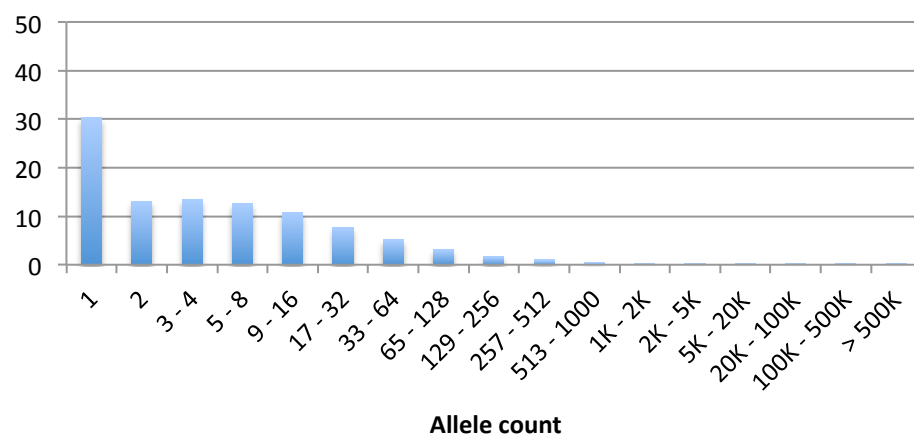

Supplement: Figure S2 — Distributions of allele frequencies and raw allele counts in simulated European-American and African-American populations. The European-American population consists almost entirely of rare variants, while the African-American population contains a wider range of rare, low-frequency, and common variants. Percentage of variants with allele frequencies and raw allele counts in the designated ranges are shown. Because of European-American allele frequencies are , we include a blow-up of frequencies , which range from 0.05% to 0.2%. Demographic models shown in Figure S11. (PDF) [file pgen.1003224.s002.pdf]

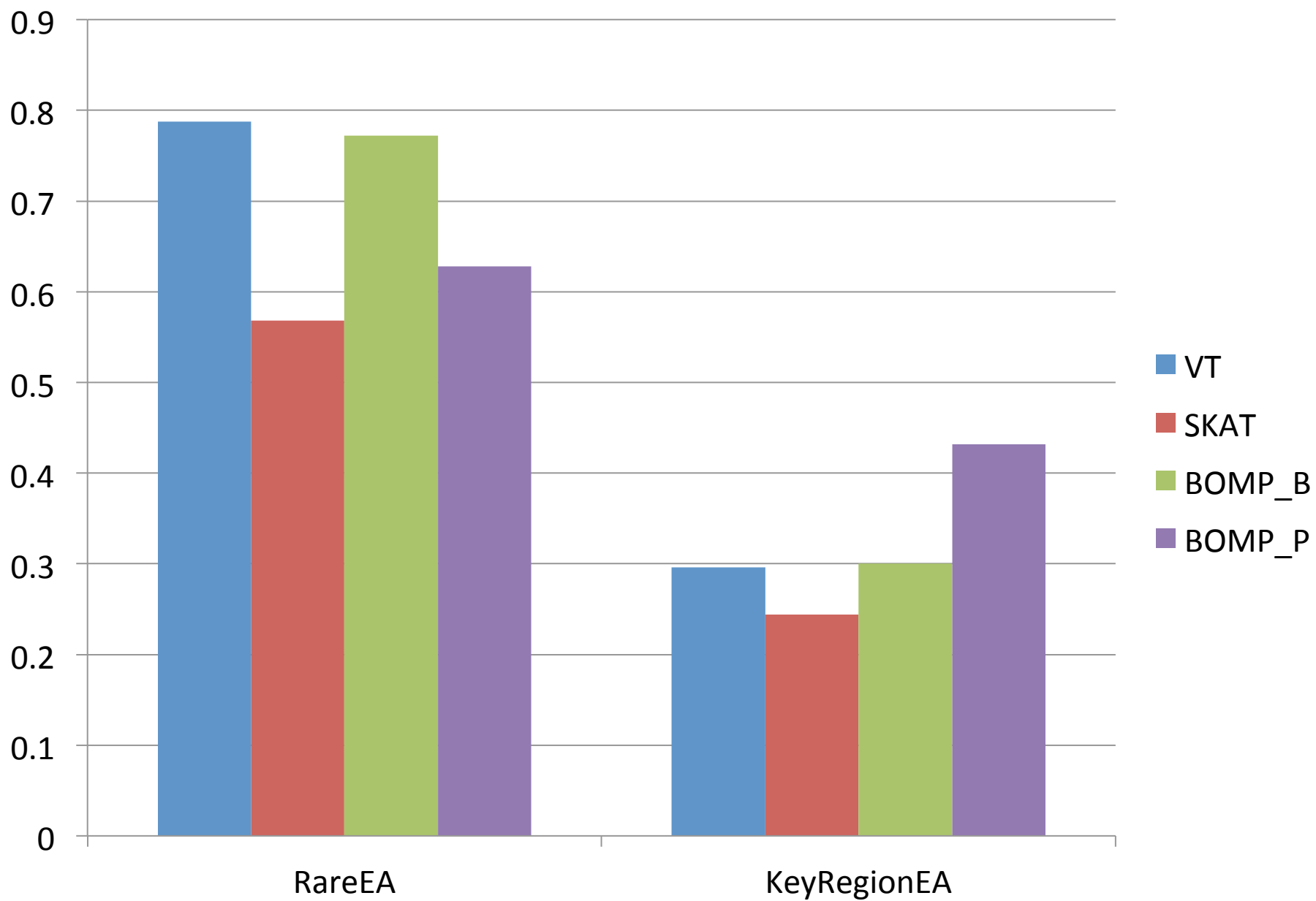

Supplement: Figure S4 — Power of position distribution statistics compared to burden methods and SKAT. Burden tests outperform the position distribution statistic when causal variants are rare and are not clustered, as in our simulations of Rare Variant disease etiology and European-American demographic. The position distribution test outperforms burden tests when the number of rare variants is similar in cases and controls, but where cases and controls differ with respect to the position distribution of the variants, as in simulations of Key Region Variant disease etiology and European-American demographic. Both collapsing burden and position distribution tests outperform SKAT when causal variants are very rare. RareEA = rare variant disease etiology (Table 1) and European-American demographic model. KeyRegionEA = key region variant disease etiology (Table 1) and European-American demographic model. Power shown on Y-axis. Simulations with 10,000 samples are shown. (PDF) [file pgen.1003224.s004.pdf]

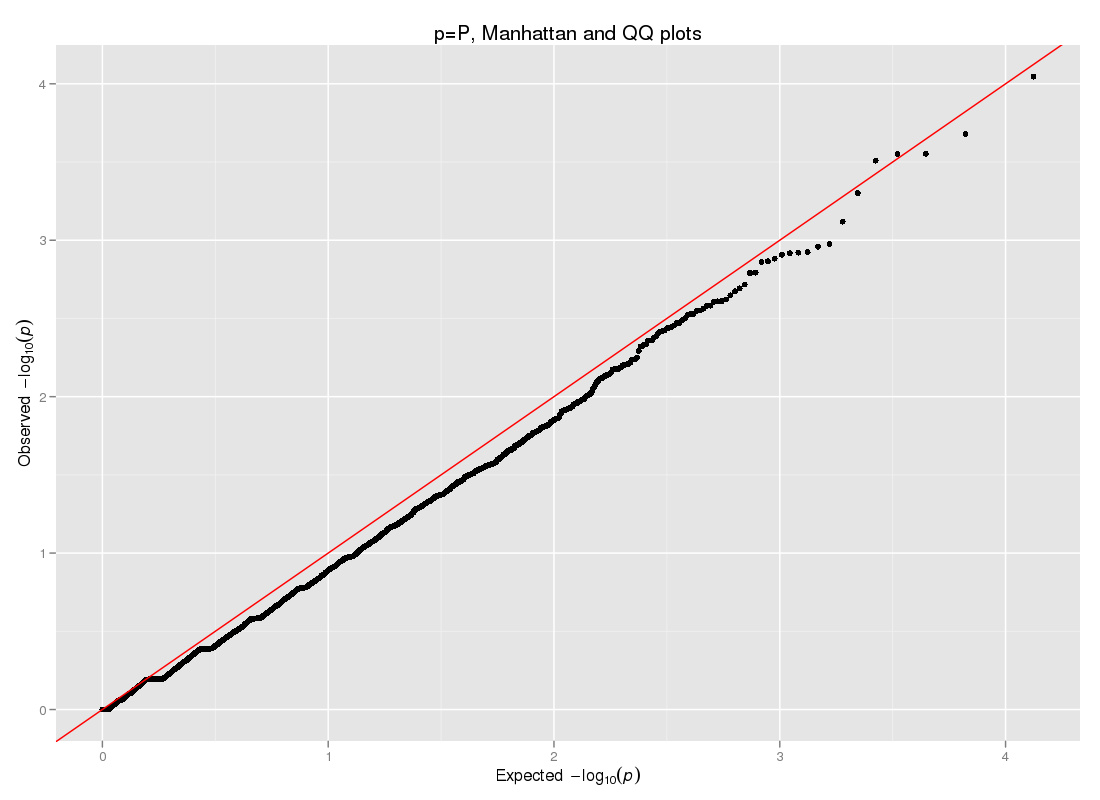

Supplement: Figure S5 — Q-Q plot of BOMP P-values for all genes in the Bipolar case-control study. The plot shows no evidence of heavy skew or heavy tails, indicating that there is no systematic bias in our analysis. Empirical P-values are below the line because the BOMP statistic is not continuous for genes with few variants in only a few samples, leading to conservative P-values. (PNG) [file pgen.1003224.s005.png]

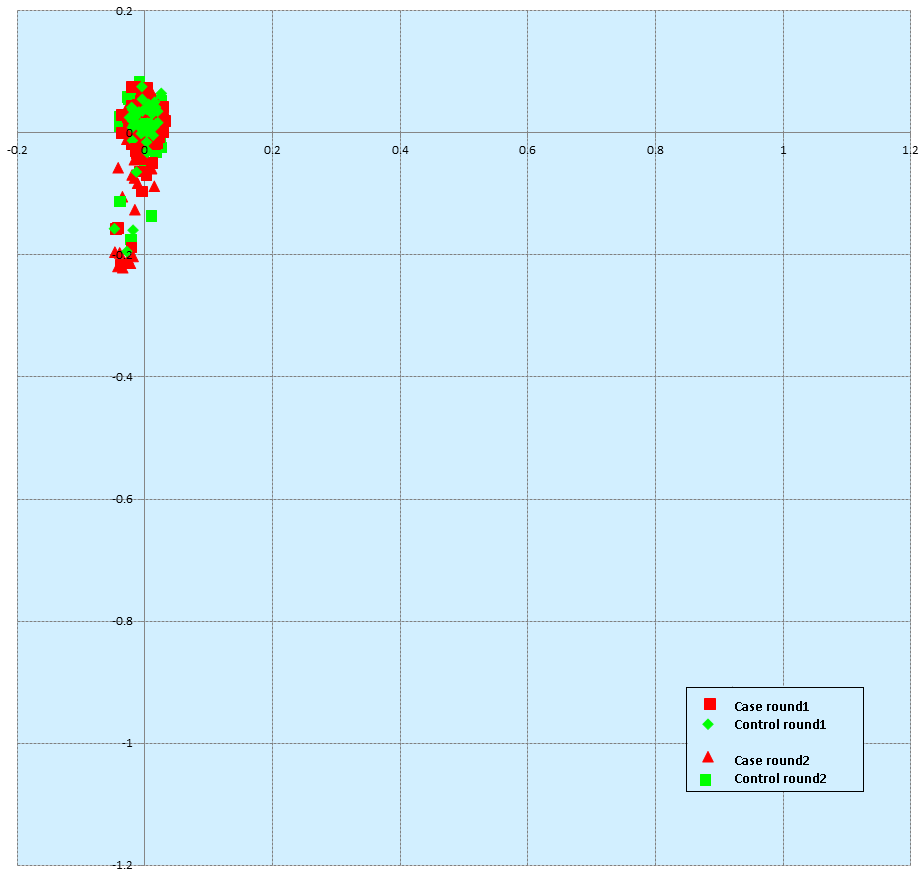

Supplement: Figure S6 — PCA plot showing the overlap of bipolar samples and controls sequenced during rounds 1 and 2. Plot obtained using EIGENSTRAT [28], where the first and second components of the model (PC1 and PC2, respectively) are shown. The analysis does not show significant differences in the nature or frequency of variants identified in the two rounds. Round 1 = Nimblegen v1.0 and Illumina GAII, Round 2 = Nimblegen v2.0 and Illumina HiSeq2000. (PNG) [file pgen.1003224.s006.png]

## cases

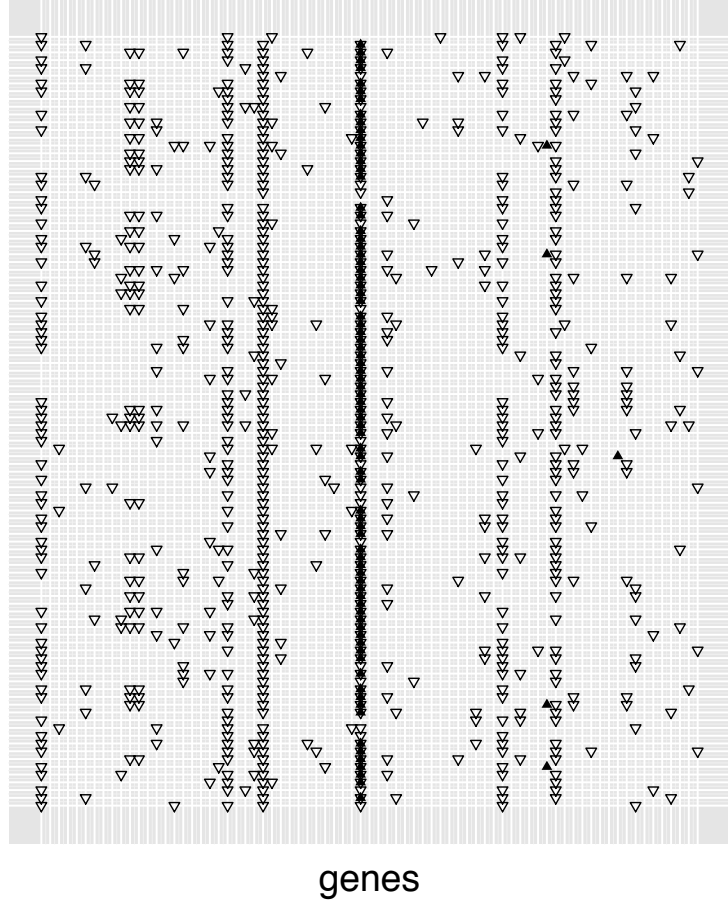

## controls

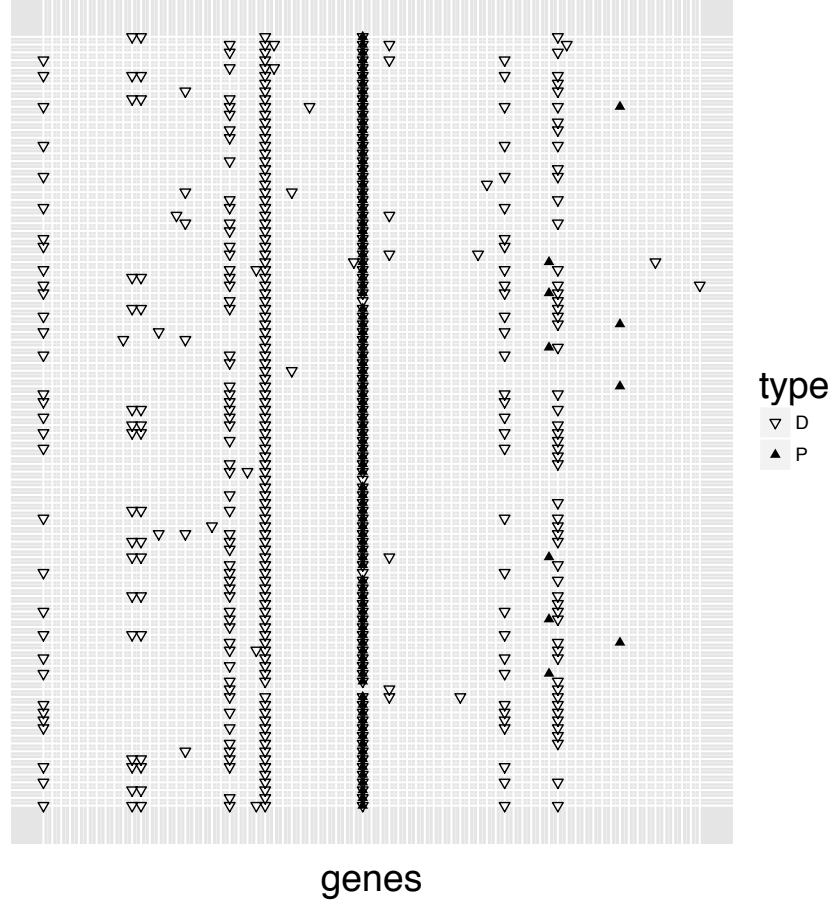

Supplement: Figure S7 — Example of how our simulations capture genetic heterogeneity in complex disease. Each horizontal grid line represents a genomic individual. (Cases and controls shown separately.) Each vertical gridline represents a gene. Causal variants (both deleterious and protective) are shown as triangles. Different case individuals have different patterns of causal variants and the allele frequencies of the variants range from rare (1 allele) to common (190 alleles). Causal variants are also observed in the control individuals. Type = Downward pointing triangles are deleterious variants, upward pointing triangles are protective variants. (200 genomic individuals from African-American demographic model are shown). (PDF) [file pgen.1003224.s007.pdf]

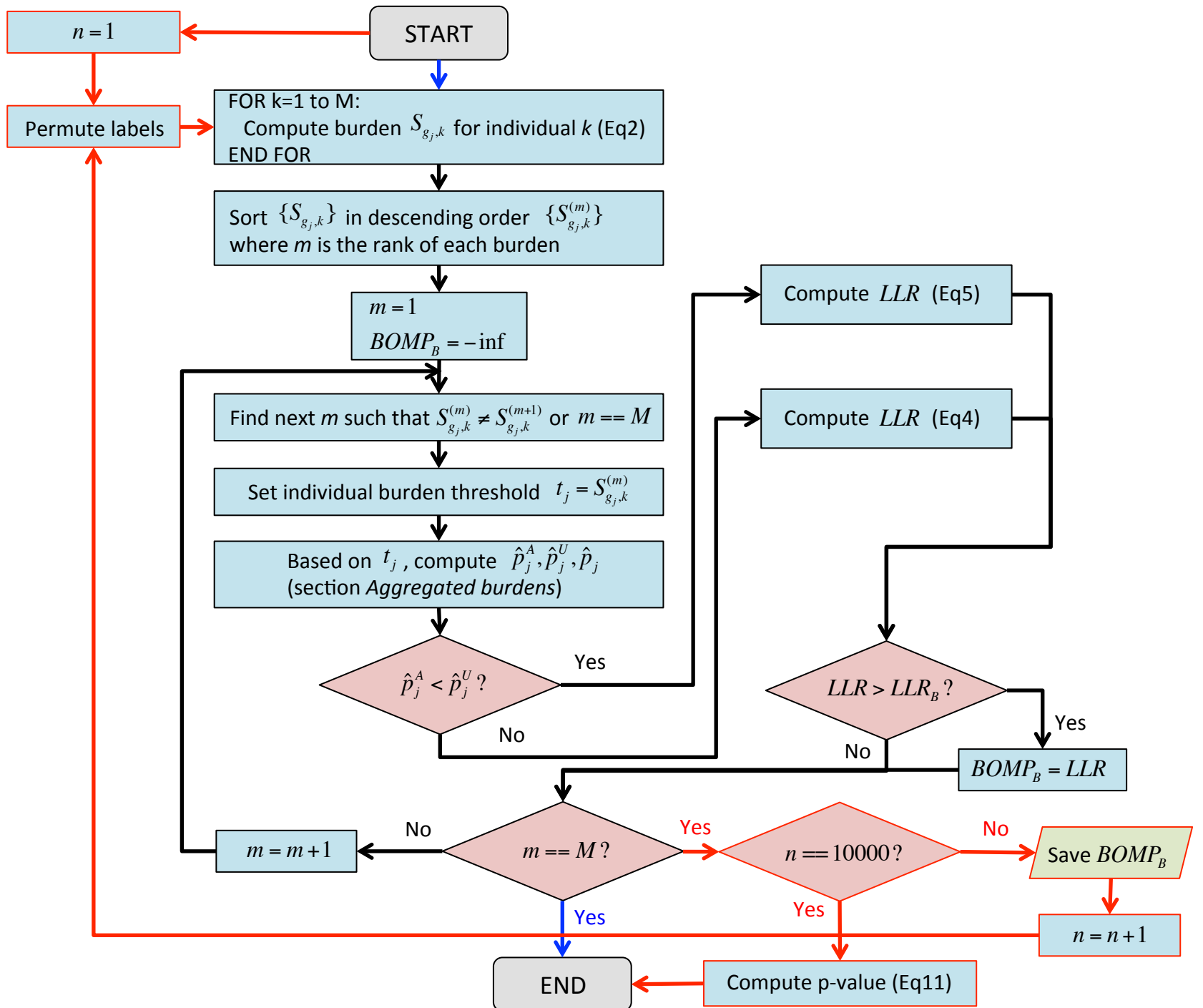

Supplement: Figure S8 — Flow chart for calculation of mutation burden statistic. The statistic is first calculated from empirical data (by following the blue and black arrows). The null mutation burden statistics are calculated by following the red and black arrows. Key steps in the calculation are: computing the burden for each individual; sorting the individual burdens into a ranked list, where denotes list rank; selecting a candidate burden threshold; computing maximum likelihood estimates of Bernoulli parameters at this threshold; computing the LLR (log-likelihood ratio) with these parameters. Candidate burden thresholds are iteratively tested and the threshold yielding the largest LLR is returned. To generate the null distribution, case-control labels are repeatedly permuted and the key steps are followed to compute one point in the null. The permuted null distribution is estimated by repeating steps 1–4 10,000 times (or user-adjusted value) and used to compute the p-value for the statistic. (PDF) [file pgen.1003224.s008.pdf]

Window Size = 8

Shift Increment = 1

**Gene**

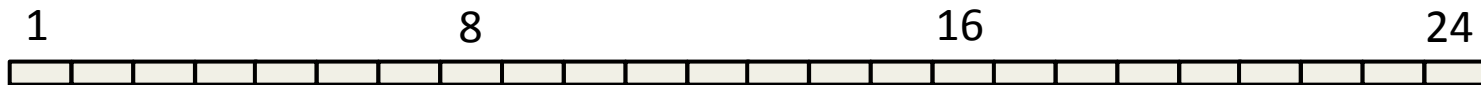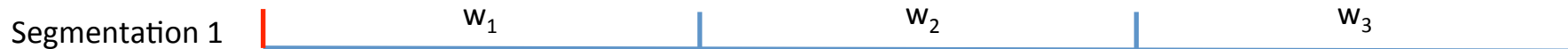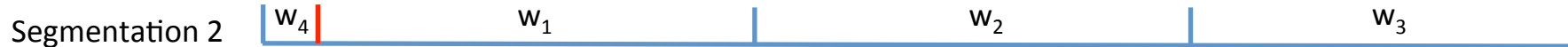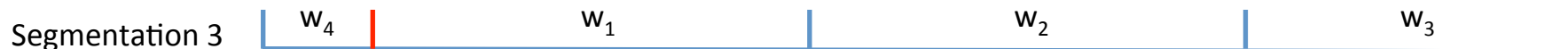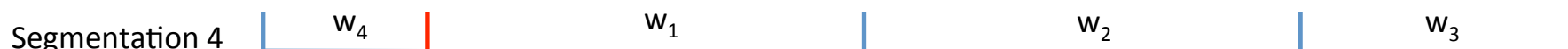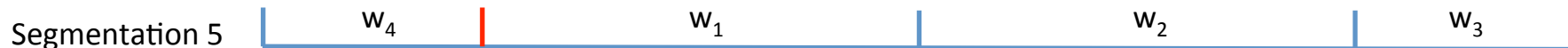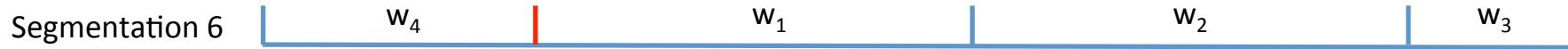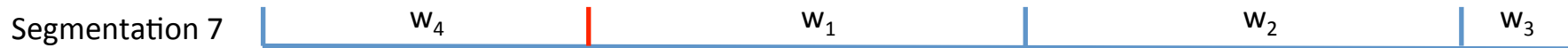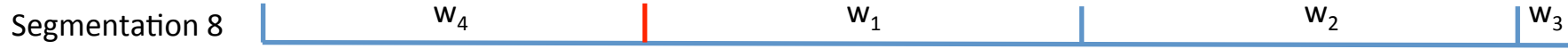

Supplement: Figure S9 — Window and sequence segmentations. The mutation position distribution statistic requires a segmentation for a sequence of interest (e.g., a gene). We generate candidate segmentations by selecting a window size and allowing a series of possible offsets, based on a selected shift increment . In this example, we illustrate the eight possible window segmentations of a gene with 24 codons (represented by rectangles), using a window size of 8 and a shift increment of 1. (PDF) [file pgen.1003224.s009.pdf]

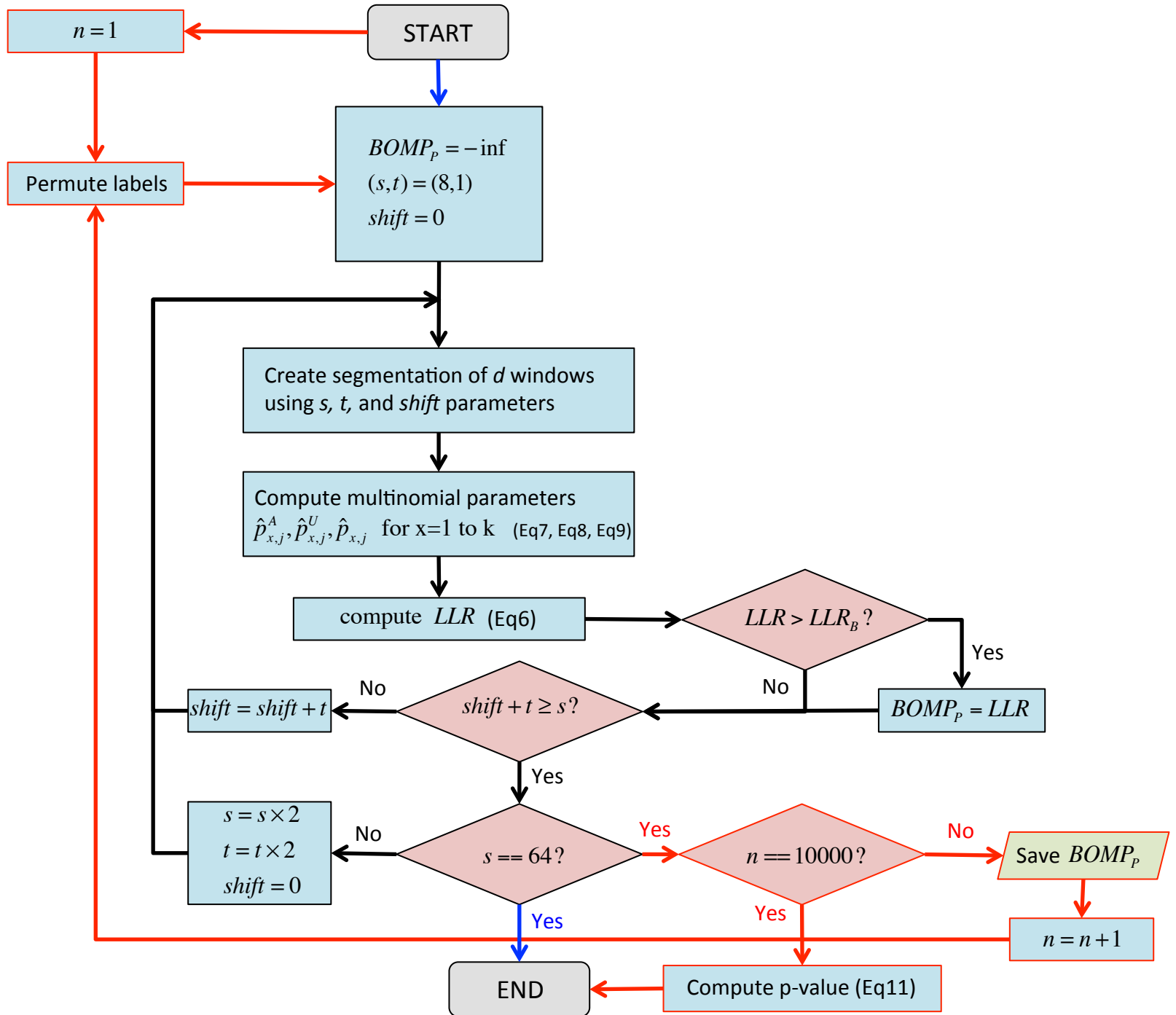

Supplement: Figure S10 — Flow chart for calculation of position distribution statistic. The statistic is first calculated from empirical data (by following the blue and black arrows). The null position distribution statistics are calculated by following the red and black arrows. Key steps in the calculation are: choice of (user-selected) window sizes and shift increment to generate a set of gene segmentations (Each unique segmentation is defined by , , and , which represents the current segmentation offset); estimation of parameters for each of three multinomial distributions – cases, controls, and cases and controls together – (for a segmentation with windows); computation of log likelihood ratio; finding the largest log likelihood ratio for all segmentations. To generate the null distribution, case-control labels are repeatedly permuted and the key steps are followed to compute one point in the null. The permuted null distribution is estimated by repeating steps 1–4 10,000 times (or user-adjusted value) and used to compute the p-value for the statistic. (PDF) [file pgen.1003224.s010.pdf]

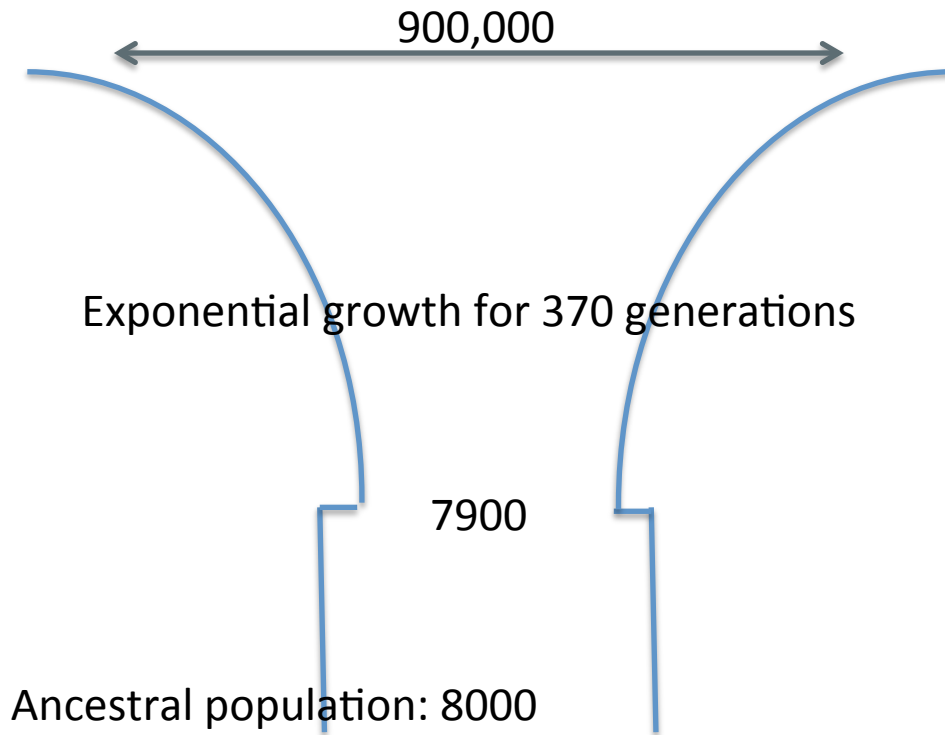

European American

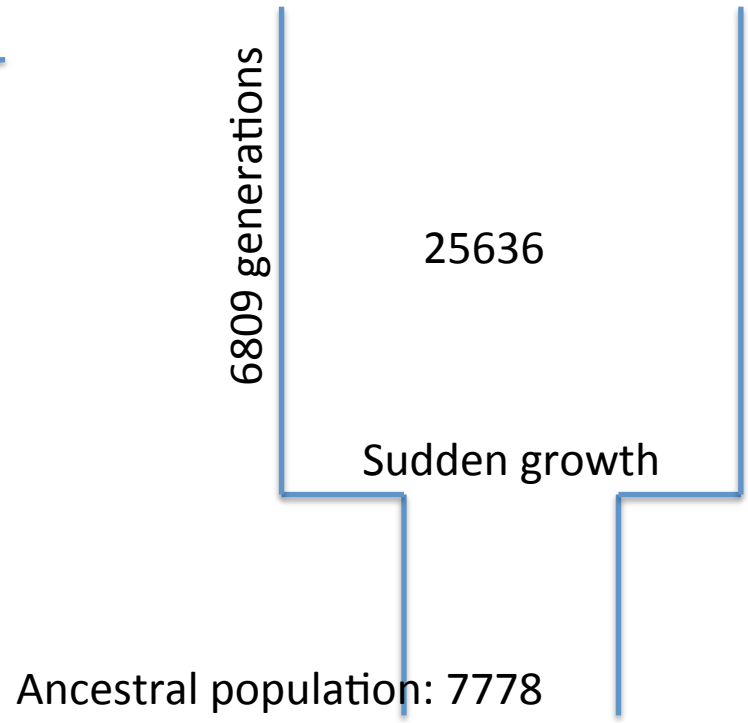

African American

Supplement: Figure S11 — Demographic models of European-American and African-American populations. The models were fit to European-American [19] and African-American sequencing data [44] (PDF) [file pgen.1003224.s011.pdf]
