# Supplementary material for: A Hybrid Likelihood Model for Sequence-Based Disease Association Studies
Source: PLoS Genet. 2013 Jan 24;9(1):e1003224. doi: 10.1371/journal.pgen.1003224 (PMC3554549; doi:10.1371/journal.pgen.1003224)
Supplement: Figure S3 — Nine multinomial distributions used to construct sets of multiple candidate genes for case-control studies. Each multinomial distribution is named for its dominant disease etiology. (PDF) [file pgen.1003224.s003.pdf]

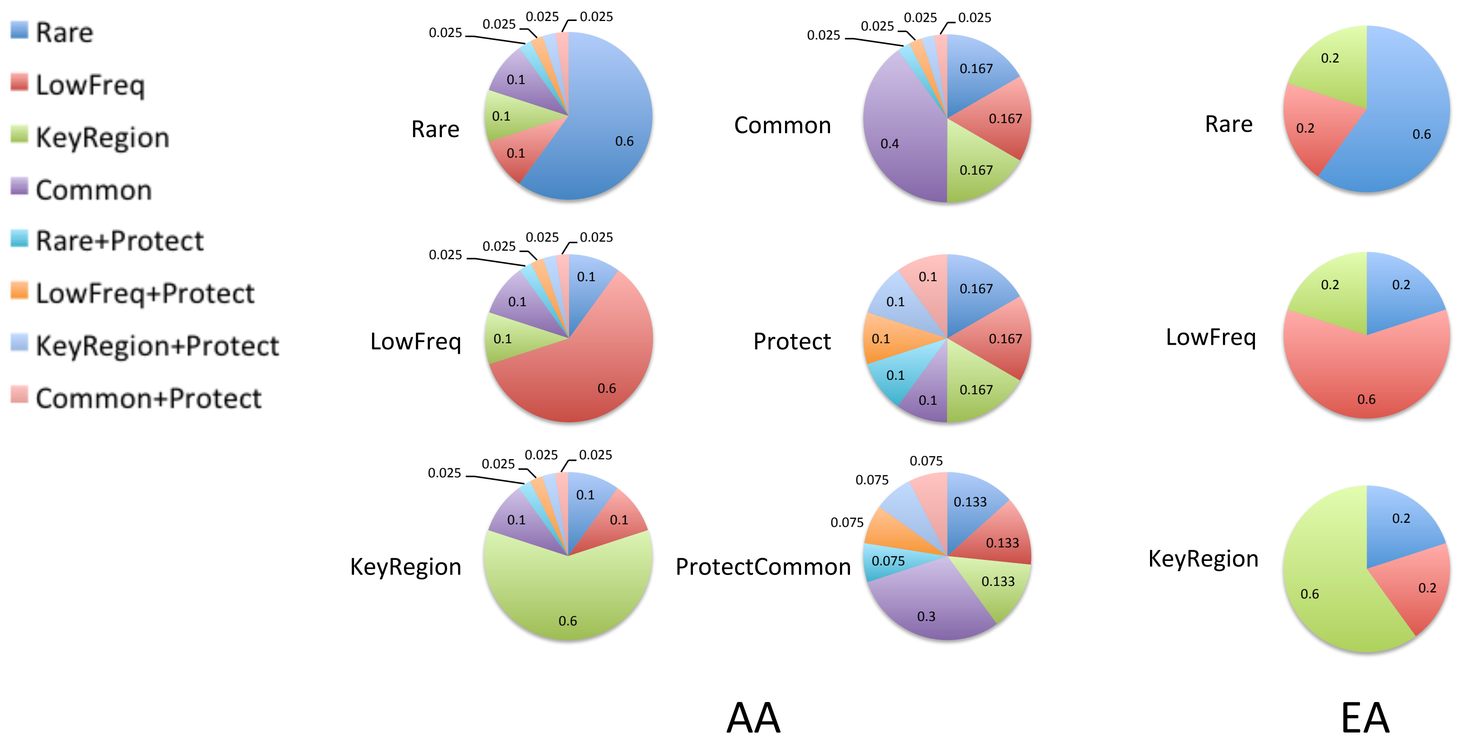
Rare
LowFreq
KeyRegion
Common
Rare+Protect
LowFreq+Protect
KeyRegion+Protect
Common+Protect
Rare
0.025
0.025
0.025
0.025
0.1
0.1
0.1
0.6
Common
0.025
0.025
0.025
0.025
0.167
0.167
0.167
0.4
Rare
0.2
0.2
0.6
LowFreq
0.025
0.025
0.025
0.025
0.1
0.1
0.1
0.6
Protect
0.1
0.167
0.1
0.167
0.1
0.1
0.167
0.1
LowFreq
0.2
0.2
0.6
KeyRegion
0.025
0.025
0.025
0.025
0.1
0.1
0.1
0.6
ProtectCommon
0.075
0.075
0.133
0.075
0.133
0.075
0.133
0.3
KeyRegion
0.2
0.2
0.6
AA
EA
