# Supplementary material for: A Hybrid Likelihood Model for Sequence-Based Disease Association Studies
Source: PLoS Genet. 2013 Jan 24;9(1):e1003224. doi: 10.1371/journal.pgen.1003224 (PMC3554549; doi:10.1371/journal.pgen.1003224)
Supplement: Table S1 — BOMP, VT, and SKAT comparison. Approaches to variant collapsing, variant importance, and choice of statistical framework define differences and similarities among BOMP, VT, and SKAT. A. Variant collapsing strategies. VT and BOMP burden both collapse variants across a genomic region. SKAT does not do collapsing and considers variants one at a time. BOMP position distribution collapses variants across local windows over a genomic region. These different collapsing strategies are illustrated in a toy example in Figure 8 of our main manuscript. B. Variant importance. All methods assume that some variants are more important than others. This idea is implemented by either filtering and/or weighting variants. C. Statistical framework. VT and BOMP burden both use a summary statistic to compare burden in cases and controls and assess the statistical significance of the statistic with permutation. For VT, individual burdens are summed over the case group and over the control group and summarized by the difference in Z-score between the two groups. For BOMP burden, individual burden is dichotomized for each sample, by selecting a burden threshold. The probability of exceeding the burden threshold for cases and for controls is estimated. The difference between the two is compared with a log-likelihood ratio. For SKAT, phenotypic labels (case or control) are directly regressed for each variant. The coefficient for each variant is tested by comparing it to 0, with a variance-component score test. Statistical significance is calculated analytically. For BOMP position distribution, the difference of distributions of variants over local windows between cases and controls is modeled by multinomial likelihood and then summarized by a log-likelihood ratio. (PDF) [file pgen.1003224.s012.pdf]

	A	B	C
	Variant Collapsing Strategies	*Variant Functional Importance*	*Statistical Framework*
**BOMP burden**	Across whole genomic region	Weighted by MAF and bioinformatics score	Likelihood ratio
**VT**	Across whole genomic region	Filtered by MAF and weighted by bioinformatics scores	Z-score
**SKAT**	No collapsing	Weighted by MAF	Regression with variance-component score test
**BOMP position distribution**	Across local window	None	Likelihood ratio
