# Supplementary material for: A Hybrid Likelihood Model for Sequence-Based Disease Association Studies
Source: PLoS Genet. 2013 Jan 24;9(1):e1003224. doi: 10.1371/journal.pgen.1003224 (PMC3554549; doi:10.1371/journal.pgen.1003224)
Supplement: Text S1 — Supplementary Material. (PDF) [file pgen.1003224.s013.pdf]

A Hybrid Likelihood Model for Sequence-Based Disease Association Studies

Yun-Ching Chen[1], Hannah Carter[1], Jennifer Parla[4], Melissa Kramer[4], Fernando S. Goes[2], Mehdi Pirooznia[2], Peter P. Zandi[2], W. Richard McCombie[4], James B. Potash[3], Rachel Karchin[1,*]

1 Department of Biomedical Engineering and Institute for Computational Medicine, Johns Hopkins University, 3400 N. Charles St., Baltimore, Maryland USA

2 Department of Psychiatry and Behavioral Sciences, Johns Hopkins School of Medicine, Baltimore, Maryland USA

3 Department of Psychiatry, University of Iowa, Iowa City, Iowa USA

4 Stanley Institute for Cognitive Genomics, Cold Spring Harbor Laboratory, Cold Spring Harbor, New York USA

∗ **E-mail: karchin@jhu.edu**

Supplementary Material

Demographic models

Kryukov *et al.* [1] used sequence data for 58 genes from 757 European-American individuals to fit parameters of a demographic model, with Wright-Fisher diffusion approximation, assuming long-term constant size succeeded by a bottleneck and then exponential growth (Figure S11). The best fitting distribution of fitness effects/selection coefficients (DFE) was a two-component mixture of gamma distributions $S \sim 0.2\text{Gamma}(1, 10^6) + 0.8\text{Gamma}(0.56231, 0.01)$. Mixture parameters and software to estimate the mixture were generously provided by G. Kryukov. The DFE does not include the possibility of protective variants influencing a phenotype of interest.

Boyko *et al.* [2] used genome-wide polymorphism data and fixed differences between the human and chimp genomes, to estimate demography and DFE for 19 African American samples, with Wright-Fisher and Poisson Random Field theory [3–5]. The best-fitting demographic model was a two-epoch instantaneous growth model for the African Americans (Figure S11). A best fitting DFE model, by maximum likelihood estimate, had three parameters: proportion of positively selected sites (1.86%); shape (0.228) and rate (16.54) of a gamma distribution for sites with deleterious fitness effects. Positive selection was fixed at $s = 9.7 \times 10^{-5}$.

Toy example with analytical calculations

Figure 7 shows a comparison of SKAT, BOMP position distribution, BOMP mutation burden, and VT burden statistics on a toy example. In this toy gene, we have genotypes of 8 cases and 8 controls at 10 positions.

SKAT does a logistic regression where phenotypic labels are the response and predictors are the genotypes (matrix $\mathbf{G}$). A variance-component score statistic Q is computed by comparing the full model with a null model, using a kernel function that incorporates allele frequency weights. The statistic follows a mixture of χ^2 distributions, enabling analytical computation of P-values.

The BOMP position distribution statistic is shown computed for a gene segmentation with d=4 windows. Window mutation counts $W^A_{x,j}$,$W^U_{x,j}$, and $W^{A+U}_{x,j}$ are computed for each window. Multinomial parameters are estimated for each window and used to compute the position distribution statistic, and P-values are estimated by permutation.

The BOMP burden statistic uses allele frequency weights (gray-scale bar) and sums weighted genotypes to compute the mutation burden of each individual. T^U_j and T^A_j are the aggregate burdens for controls and cases, respectively. $\hat{p}^A_j$, $\hat{p}^U_j$, and $\hat{p}_j$ are estimates of the probability that a case will exceed the burden threshold, that a control will exceed the burden threshold, and that any individual in the study will exceed the burden threshold. They are used as parameters of three Bernoulli distributions (one for cases, one for controls, and one for cases and controls together). These parameters are used to compute the BOMP burden statistic, and P-values are estimated by permutation. The final BOMP statistic is the sum of its burden and position distribution statistics (P-values estimated by permutation).

The VT burden test selects an optimal MAF threshold, by trying many thresholds and identifying the one that maximizes the z-score difference between cases and controls. All variants that exceed the threshold are filtered out and not considered for analysis. The remaining variants are summed in cases and controls and a z-score statistic is used to quantify their difference. P-values are estimated by permutation.

Disease etiologies and demographic models affect method performance

In our simulations, we found that the performance of all tested methods depended on choice of disease etiology and demographic model.

The VT method is a burden test, a class of methods in which it is assumed that all variants lower than a MAF of interest are deleterious. Burden tests accumulate signal by collapsing variants across a genomic region. They are valuable in detecting associations between rare variants and disease phenotypes, when case-control studies are not large enough to detect association between a single rare variant and disease. An important advance of the VT burden test is that it adaptively learns a MAF threshold from the data, efficiently filtering out a large number of non-causal variants. In our simulations, it always has the most power for rare variant etiology and European-American demographic, in which the population is enriched for very rare variants. However, in other etiologies, it does not do quite as well. For example, in the low frequency etiology, in which causal variants must have at least 0.1% MAF, a single MAF threshold will not well separate causal and non-causal variants. VT also loses some power when common or protective variants are included in an etiology and does not gain power when variant positions are distributed differently in cases and controls.

The SKAT linear kernel regression model circumvents an important limitation of burden tests, which is that they can only detect causal variants that are deleterious. In contrast, SKAT belongs to a class of methods in which emphasis is placed on the variance of genotype frequencies in cases and controls. It does no collapsing and each variant (genotype) is treated as an independent covariate. Thus, it is sensitive to both deleterious and protective effects, and it has good power in the presence of protective variants. SKAT weights variants according to their MAF, by treating the MAF as a random variable from a Beta density. Beta parameters are set so as to give increased weight to those MAFs considered most likely to be causal *a priori*. Using default settings, common variants get decreased weight, which is reflected in power loss for our common variant etiology (MAF>5%). Variants in our low frequency etiology (0.1%<MAF≤5%) are well captured by this Beta density, and we see that SKAT has good power for this etiology. While rare variants also get high SKAT weights, we found that SKAT loses power for our rare variant etiology in the European-American demographic model. Because there are many extremely rare variants present, the SKAT model will estimate a very small marginal effect size for each variant and fail to reject the null hypothesis of zero marginal effect size.

KBAC differs from SKAT, VT, and BOMP in that the phenotype association of a genotype vector, rather than single variants, is calculated. Each individual is represented by a pattern of 0's, 1's, and 2's for M sites in a candidate genomic region. Only sites where rare variants have been seen in a case or

control are included. An adaptive weight is computed for each vector using a mixture model, and the KBAC statistic for a genomic region of interest is the weighted sum of vector frequency differences in cases vs. controls. A strength of this strategy is that it implicitly considers interactions among variants, which are not incorporated into the other tested methods. A weakness is that the information in a vector frequency difference depends on the number of individuals sharing a common vector. In sequencing studies with many rare variants, the probability of seeing the same vector more than once is low. In our simulations, there are no interactions among variants and an important strength of KBAC is not utilized. Particularly in simulations involving the European-American exponential demographic model, which is enriched for very rare variants and thus has few shared vectors, KBAC has low power. Like SKAT, KBAC can handle protective variation, and in our simulations, it has relatively good power for etiologies that include protective variants. KBAC only considers variants with less than a pre-specified MAF to be causal. We selected MAF thresholds of (recommended) 1% and 5%, which resulted in KBAC having low power for our common variant etiology. However, KBAC can be extended to include common variants as co-variates in a logistic regression model, which could improve power for this etiology.

BOMP is a hybrid likelihood model, which conceptually (but not mathematically) incorporates the general approaches represented by VT and SKAT. VT assumes that cases have more variants in phenotype-associated genomic regions, with MAF below an optimized threshold, than controls. SKAT assumes that the subset of variants that are truly phenotype-associated are over-represented in cases or controls. The BOMP burden statistic captures the scenario in which cases have more variants than controls (allele frequencies are incorporated via coefficient scores), while the position distribution statistic captures the scenario in which cases have more variants than controls (or vice versa) in highly localized, functionally important genomic regions, from 8 to 64 codons in length. Essentially, SKAT does the same thing, except that the localization unit is defined as a single variant (codon or nucleotide). When SKAT or BOMP position distribution statistic detect significant differences between cases and controls within a localization unit, no further collapsing takes place. Thus, both do well at detecting protective variants. However, using localized collapsing, the BOMP position distribution statistic gains power to detect differences between cases and controls (Figure 6) that would be missed by either SKAT or a burden statistic.

BOMP's combination statistic is effective for most of our tested etiologies. It is particularly effective for the key region etiology, but relatively less effective for the rare variant etiology, particularly with the European-American demographic. For this etiology, by construction, the causal rare variants are distributed randomly across the simulated genomic region, and the BOMP position distribution statistic gains no power by collapsing within a localized region. A burden statistic using an optimized MAF threshold is very effective for this etiology.

Algorithm complexity

BOMP burden

For each gene, computing the BOMP burden statistic is $O(MlogM)$ where M is the number of samples. As shown in Figure S8, first, we loop over M samples and for each sample we sum over the variants in that sample. The matrix of variants vs. samples is sparse, thus summing over the number of variants for M samples is proportional to M. Next, we perform an $MlogM$ sort. Finally, we maximize the likelihood ratio by iterating over the individual burdens computed for each sample, which is also proportional to M. All other operations in the optimization take constant time (including estimates of Bernoulli parameters and log likelihood ratios). The final complexity is $O(MlogM)$.

BOMP position distribution

For each gene, computing the position distribution statistic is $O(M) + O(L)$, where M is the number of samples and L is the length of the gene. The total calculation is for a constant number of segmentations. For each segmentation, we project all variants onto a set of windows, in time proportional to the total number of variants. Given the sparsity of the variants vs samples matrix, the total number of variants is proportional to the total number of samples M. Finally, we maximize the likelihood ratio by estimating parameters for each window, which is proportional to the length of the gene L (Figure S10). The final complexity is $O(M) + O(L)$.

In practice, the algorithms performance is often better than predicted by theory, as shown in our benchmarks (Computational Time section in main manuscript).

Effect size of common variant

Most significant common variants in GWAS have odds ratios < 2.0 [6]. For case-control simulations, an odds ratio was analytically equated with mean shift in a quantitative phenotypic trait, given the assumed disease prevalence of 1%. Effect size in disease etiology "common" (Table 1) was 0.1σ, equivalent to an odds ratio of 1.41. The equivalence can be shown as follows:

Let a causal variant have a mean shift of 0.1σ and an allele frequency of 0.1 in a case-control study. Let X be the value of the trait when no causal variant is present in an individual. Let Y be the value of the trait when a causal variant is present in an individual.

$$X \sim N(0, 1)$$

$$Y \sim N(0.1, 1)$$

Then density of trait values in the population under study can be calculated from the linear combination of these two variables:

$$0.9X + 0.1Y \sim N(0.9 \times 0 + 0.1 \times 0.1, 0.9^2 + 0.1^2) = N(0.01, 0.82) \tag{1}$$

The cutoff for the affected population (and cases) is then $Q(0.99) = 1.92$ and the cutoff for the unaffected population (and controls) is $Q(0.25) = -0.54$, where Q is the quantile function of $N(0.01, 0.82)$. Then the odds ratio for is:

$$\frac{P(\text{case}|V = 1)/P(\text{control}|V = 0)}{P(\text{control}|V = 1)/P(\text{control}|V = 0)} = \frac{1 - F_{\text{causal}}(1.92)/1 - F_{\text{no\_causal}}(1.92)}{F_{\text{causal}}(-0.54)/F_{\text{no\_causal}}(-0.54)} = 1.41 \tag{2}$$

where

$$V = \begin{cases} 1 & \text{if an individual carries V;} \\ 0 & \text{otherwise.} \end{cases}$$

and F_{causal} is the CDF of $N(0.1, 1)$ and $F_{\text{no\_causal}}$ is the CDF of $N(0, 1)$.

References

1. Kryukov GV, Shpunt A, Stamatoyannopoulos JA, Sunyaev SR (2009) Power of deep, all-exon resequencing for discovery of human trait genes. Proc Natl Acad Sci U S A 106: 3871–3876.

2. Boyko AR, Williamson SH, Indap AR, Degenhardt JD, Hernandez RD, et al. (2008) Assessing the evolutionary impact of amino acid mutations in the human genome. PLoS Genet 4: e1000083.

3. Sawyer SA, Hartl DL (1992) Population genetics of polymorphism and divergence. Genetics 132: 1161–1176.

4. Bustamante CD, Wakeley J, Sawyer S, Hartl DL (2001) Directional selection and the site-frequency spectrum. Genetics 159: 1779–1788.

5. Bustamante CD, Nielsen R, Hartl DL (2003) Maximum likelihood and bayesian methods for estimating the distribution of selective effects among classes of mutations using dna polymorphism data. Theor Popul Biol 63: 91–103.

6. Bodmer W, Bonilla C (2008) Common and rare variants in multifactorial susceptibility to common diseases. Nat Genet 40: 695–701.
